# Characterization of *Clostridioides difficile* DSM 101085 with A^−^B^−^CDT^+^ Phenotype from a Late Recurrent Colonization

**DOI:** 10.1093/gbe/evaa072

**Published:** 2020-04-17

**Authors:** Thomas Riedel, Meina Neumann-Schaal, Johannes Wittmann, Isabel Schober, Julia Danielle Hofmann, Chia-Wen Lu, Antonia Dannheim, Ortrud Zimmermann, Matthias Lochner, Uwe Groß, Jörg Overmann

**Affiliations:** e1 Leibniz Institute DSMZ-German Collection of Microorganisms and Cell Cultures, Braunschweig, Germany; e2 German Center for Infection Research (DZIF), Partner Site Hannover-Braunschweig, Braunschweig, Germany; e3 Department of Bioinformatics and Biochemistry and Braunschweig Integrated Centre of Systems Biology (BRICS), Technische Universität Braunschweig, Germany; e4 Institute of Infection Immunology, TWINCORE, Centre for Experimental and Clinical Infection Research, a Joint Venture between the Medical School Hannover (MHH) and the Helmholtz Centre for Infection Research (HZI), Hannover, Germany; e5 Institute of Medical Microbiology, University Medical Center Göttingen, Germany; e6 Göttingen International Health Network, Göttingen, Germany; e7 Institute of Microbiology, Technical University of Braunschweig, Germany

**Keywords:** *Clostridioides difficile*, *Clostridium difficile*, pathogenicity locus, binary toxin, fermentation profile, R20291 resequencing

## Abstract

During the last decades, hypervirulent strains of *Clostridioides difficile* with frequent disease recurrence and increased mortality appeared. *Clostridioides difficile* DSM 101085 was isolated from a patient who suffered from several recurrent infections and colonizations, likely contributing to a fatal outcome. Analysis of the toxin repertoire revealed the presence of a complete binary toxin locus and an atypical pathogenicity locus consisting of only a *tcdA* pseudogene and a disrupted *tcdC* gene sequence. The pathogenicity locus shows upstream a transposon and has been subject to homologous recombination or lateral gene transfer events. Matching the results of the genome analysis, neither TcdA nor TcdB production but the expression of *cdtA* and *cdtB* was detected. This highlights a potential role of the binary toxin *C. difficile* toxin in this recurrent colonization and possibly further in a host-dependent virulence.

Compared with the *C. difficile* metabolic model strains DSM 28645 (630*Δerm*) and DSM 27147 (R20291), strain DSM 101085 showed a specific metabolic profile, featuring changes in the threonine degradation pathways and alterations in the central carbon metabolism. Moreover, products originating from Stickland pathways processing leucine, aromatic amino acids, and methionine were more abundant in strain DSM 101085, indicating a more efficient use of these substrates. The particular characteristics of strain *C. difficile* DSM 101085 may represent an adaptation to a low-protein diet in a patient with recurrent infections.

## Introduction

*Clostridioides difficile* is a major nosocomial human pathogen causing diarrhea in patients with a compromised gut microbiota due to treatment with broad-spectrum antibiotics (Lessa et al. 2015). In 2016, *Clostridium difficile* (Hall and O’Toole 1935) Prévot 1938 was reclassified as *C. difficile* (Hall and O’Toole 1935) (Lawson et al. 2016). The number of community-acquired infections and cases of young and healthy individuals has been rising over the past decades (Lessa et al. 2015). The symptoms of *C. difficile* infections (CDIs) can range from relatively mild diarrhea to pseudomembranous colitis and toxic megacolon (Rupnik et al. 2009; Knight et al. 2015). Symptoms of CDI are considered to be associated with the production of two toxins, Toxin A (TcdA) and toxin B (TcdB) (Kuehne et al. 2010). These toxins glycosylate and consequently inactivate Rho-family GTPases after uptake into the host cells leading to the disruption of the cytoskeleton, apoptosis, and a strong inflammatory response (Jank and Aktories 2008). Toxin A and toxin B are encoded by a 19.6-kb chromosomal region known as the pathogenicity locus (PaLoc) (Braun et al. 1996). In addition to the toxin-encoding genes *tcdA* and *tcdB*, the PaLoc contains four accessory genes: *tcdR* and *tcdC*, which encode proteins involved in transcriptional regulation (Mani and Dupuy 2001; Matamouros et al. 2007; Bakker et al. 2012; Cartman et al. 2012), *tcdE*, which encodes a holin-like protein related to bacteriophages required for efficient secretion of the toxins (Tan et al. 2001; Govind and Dupuy 2012), as well as a gene encoding the hypothetical protein TcdL (located between *tcdE* and *tcdA*) that is associated with a *N*-acetylmuramoyl-l-alanine amidase (Monot et al. 2011; Dannheim, Riedel, et al. 2017; Mehner-Breitfeld et al. 2018). The PaLoc is often detected in the same genomic location and is replaced by a highly conserved 115- or 75-bp noncoding region in nontoxigenic strains (Dingle et al. 2014) but can also be integrated in different genomic locations that are distant from the classical PaLoc integration site (Janezic et al. 2015; Monot et al. 2015).

Some *C. difficile* isolates also produce a binary *C. difficile* toxin (CDT) (Gerding et al. 2014; Aktories et al. 2018). Based on phylogenomic analysis and multilocus sequence typing, the species *C. difficile* currently encompasses up to eight clades (Ramírez-Vargas et al. 2018). The CDT is only found in representatives of clades 2, 3, 5, as well as C-I (Dingle et al. 2014; Elliott et al. 2014, 2017; Knight et al. 2015; Janezic et al. 2016; Riedel, Wetzel, et al. 2017; Ramírez-Vargas et al. 2018). Some CDI cases with severe disease caused by the ribotype RT033 of clade 5 were reported in France but in routine diagnosis, RT033 is rarely identified (Eckert et al. 2015). Furthermore, RT033 and the sequence type (ST) ST11 lineage, in general, have origins in livestock in Europe, Asia, and Australia (Knight and Riley 2016). Recently, RT033 has also been identified as major cause of CDI in Czech horses (Kecerova et al. 2019).

The CDT toxin consists of two polypeptides: the active component CDTa (an actin-specific ADP ribosyltransferase) and the binding component CDTb (Perelle et al. 1997; Schwan et al. 2009; Papatheodorou et al. 2011). CDT is encoded by the two genes *cdtA* and *cdtB*, which are located in one operon in the binary toxin locus (CdtLoc) mostly on the chromosome. Recently, the CdtLoc was additionally found to be encoded on extrachromosomal replicons (Riedel, Wittmann, et al. 2017; Ramírez-Vargas et al. 2018). The 6.2-kb CdtLoc encodes a response regulator gene *cdtR*, which activates CDT production (Carter et al. 2007) and also affects the expression of *tcdA* and *tcdB* (Lyon et al. 2016). In *C. difficile* strains that do not produce CDT, the CdtLoc is replaced by a 68-bp noncoding region or consists of truncated and disrupted genes (Carter et al. 2007; Gerding et al. 2014; Riedel, Wetzel, et al. 2017). The role of CDT in infection and disease is still a matter of discussion because many virulent strains do not produce CDT. However, it has been shown that CDT depolymerizes the host cytoskeleton and enhances the adhesion of *C. difficile* cells to the epithelium via the formation of microtubule-based protrusions by the host cell (Schwan et al. 2011; Aktories et al. 2012). Moreover, it was shown that CDT can serve as a vehicle for intracellular delivery of bacterial glycosyltransferase domains (Beer et al. 2018) and that CDT contributes to virulence by partially suppressing the host immune response (Cowardin et al. 2016).

Toxin A and B levels detectable in stool samples are correlated with the severity of CDI (Åkerlund et al. 2006). Toxin expression is linked to metabolic activity and is initiated upon entry into the stationary growth phase (Dupuy and Sonenshein 1998). The production of toxins is influenced by growth conditions such as growth-limiting concentrations of biotin or the presence of short chain organic acids, sugars, or certain amino acids (Martin-Verstraete et al. 2016). Previous studies also showed that substrates of Stickland pathways and of the central carbon metabolism can increase or decrease toxin production, indicating a complex regulatory pattern (Dupuy and Sonenshein 1998; Karlsson et al. 2008; Bouillaut et al. 2013).

Recently, we analyzed 19 *C. difficile* isolates for the stationary phase exometabolome with one metabolic outstanding isolate, strain *C. difficile* DSM 101085 (Riedel, Wetzel, et al. 2017). In the present study, we characterized this isolate in detail in its actively growing state for its metabolic features in comparison to the well-studied reference strains DSM 28645 (630Δ*erm*) and DSM 27147 (R20291) and integrated available genomic features and metadata into our analysis. Strain *C. difficile* DSM 101085 is a member of clade 5 (Riedel, Wetzel, et al. 2017) and the ST11. In addition, strain DSM 101085 encodes a CdtLoc and an atypical PaLoc. Our genome data indicate that this isolate has evolved from the previous reinfection over time (compared with isolate 10, Sachsenheimer et al. 2018) and that it comprises a unique toxin pattern in combination with unusual metabolic features that likely represent specific adaptations to its human host.

## Materials and Methods

### Strains, Metadata, and Cultivation Conditions

*Clostridioides difficile* strains DSM 101085 (clade 5, Riedel, Wetzel, et al. 2017), DSM 27147 (R20291, RT027, clade 2, Stabler et al. 2009), and DSM 28645 (630Δ*erm*, RT012, clade 1, originally described by Hussain et al. [2005], resequenced by Dannheim, Riedel, et al. [2017] and Dannheim, Will, et al. [2017], for a comparison see Roberts and Smits [2018]) were used for our comparative analysis. Strain DSM 101085 was the final isolate from a patient (age: 70–75) with end-stage chronic kidney disease and congestive heart failure who eventually succumbed to his diseases during the course of the CDI. It was isolated from a sample during an acute episode of diarrhea following 43 days after the previous CDI episode. Eight previous episodes of CDI ranging from mild to severe symptoms separated by 4–14 weeks without symptoms, four treatments with vancomycin once combined with fidaxomycin and further details of the course of underlying diseases were described by Sachsenheimer et al. (2018). Due to his kidney disease, the patient was on a protein reduced diet. *Clostridioides difficile* could not be detected in persons in contact with the patient. Approval for this study was obtained from the Ethics Committee of the University Medical Center Göttingen (ID of Ethic Committee approval: 11/4/15).

For quantification of toxin levels, cells of all three strains were grown in parallel in a casamino acids-containing defined medium (Neumann-Schaal et al. 2015) at 37 °C and harvested after 24 h for quantitative polymerase chain reaction (PCR) or 48 h for extracellular quantification of TcdA and TcdB. For metabolome and exometabolome analysis, cells were grown in the same medium to mid-log phase and harvested anaerobically by centrifugation (10 min, 10,000 rpm, 4 °C) using gas-tight polypropylene tubes (Trasadingen, Switzerland). The supernatant for extracellular metabolome analysis was sterile filtered and frozen at −80 °C. After removing the supernatant, the precipitated cells were immediately quenched in precooled isotonic sodium chloride/methanol (50% [v/v], −32 °C) by resuspension. To remove the quenching solution, the cells were centrifuged again (5 min, 10,000 rpm, −20 °C). The precipitated cells for intracellular metabolome analysis were frozen in liquid nitrogen.

### Genome Sequencing of *C. difficile* DSM 27147 and Genome Analysis

The complete and closed genomes of the strains DSM 101085 and DSM 28645 (630Δ*erm*) were previously sequenced by a combination of single-molecule real-time and Illumina sequencing technology (Dannheim, Riedel, et al. 2017; Dannheim, Will, et al. 2017; Riedel, Wetzel, et al, 2017). Strain DSM 27147 (R20291) was resequenced within this study employing the same sequencing technology combination to ensure the identity of the strain deposited at the DSMZ and for a detailed comparison to the reference strains. Genome sequencing and genome assembly were carried out as previously described (Riedel, Wetzel, et al. 2017). For the PacBio long-read assembly of strain DSM 27147 (R20291), 71,879 postfiltered reads with an average read length of 8,388 bp were used. The resequenced genome (GenBank accession number CP029423.1) was compared with the previously existing R20291 genome sequence (He et al. 2010); all data are summarized in supplementary material 1 and supplementary table 1, Supplementary Material online. In addition, the complete genomes of strain DSM 101085 (GenBank accession numbers CP021319.1 and CP021320.1, Riedel, Wetzel, et al. 2017), DSM 27147 (GenBank accession number CP029423.1), and DSM 28645 (GenBank accession numbers CP016318.1 and CP016319.1, Dannheim, Riedel, et al. 2017) were used for genomic comparison and analysis (supplementary material 2, Supplementary Material online). Orthologous proteins were determined with ProteinOrtho (Lechner et al. 2011) (supplementary table 2, Supplementary Material online).

**Table 1:** Identified Transposon Sequences and Prophage Regions

Transposon/ Prophage ID	Start Position	End Position	Size (kb)	GC %	CDS	Transposon-Associated Genes	BlastN Result (Highest Score)	Query Cover (%)	Identity (%)
TP1	262,248	283,036	20.8	44.5	23	Transposase gene (SR_TndX_transposase cd03770), transposon-encoded protein TnpW	CTn*6*-like	62	96
TP2	379,630	387,183	7.6	35.7	9	Integrase gene (INT_ICEBs1_C_like cd01189 and Phage_integrase pfam00589), excisionase gene (Tn916-Xis pfam09035)	Tn*6218*-like (strain Ox746b, HG002386.1)	100	98
TP3	671,671	682,087	10.4	34.3	12	Integrase gene (INT_ICEBs1_C_like cd01189 and Phage_integrase pfam00589), excisionase gene (Tn916-Xis pfam09035)	Tn*6218*-like (strain Ox2167, HG002396.1)	44	91
TP4	1,687,598	1,709,187	21.6	41.2	25	Integrase gene (Phage_integrase pfam00589 and recomb_XerD TIGR02225), excisionase gene (Tn916-Xis pfam09035)	Tn*6194*-like (strain CII7, HG475346.1)	84	89
TP5	1,897,526	1,909,963	12.4	37	15	Integrase gene (XerD COG4974 and INT_ICEBs1_C_like cd01189), excisionase gene (Tn916-Xis pfam09035)	Tn*6218*-like (Ox42, HG002387.1)	37	95
PT	1,356,873	1,384,689	27.3	27.9	32	—	phiCD481-1 (LN681538.1)	12	76
PR1	2,036,124	2,108,944	72.8	27.6	126	—	phiCD506 (LN681540.1)	12	92
PR2	3,159,637	3,211,581	51.9	28.6	82	—	phiMMP03 (LN681542.1)	51	98

In addition, the genome of strain DSM 101085 was uploaded to the *Clostridioides* database in EnteroBase (http://enterobase.warwick.ac.uk) and classified by single-linkage hierarchical clustering as described previously (Frentrup et al. 2019; Zhou et al. 2020). By using single-linkage hierarchical clustering within EnteroBase (Frentrup et al. 2019; Zhou et al. 2020), the genome sequence from DSM 101085 was classified as HC150|375. This HC150 clade also contains four previously published genome sequences from PCR ribotype RT033 isolates from Australia (RPH0101), the UK (IS58, C00002448), and Slovenia (OCD52) (see http://enterobase.warwick.ac.uk). Because hierarchical clusters at the HC150 level commonly correlate with PCR ribotyping (Frentrup et al. 2019), we conclude that DSM 101085 may also be affiliated with RT033 or with a closely related ribotype.

For genome comparison of strain DSM 101085 with the previous strain isolated from the patient (isolate 10, Sachsenheimer et al. 2018), Illumina reads of isolate 10 were mapped on the complete genome of strain DSM 101085 with the Burrows–Wheeler transform (Li and Durbin 2009) followed by subsequent automatic detection of sequencing errors by Varscan (http://varscan.sourceforge.net) and GATK Consensus calling (https://software.broadinstitute.org/gatk/). In case of nucleotide variants, the majority fraction was called for the final consensus sequence, which was manually approved by using the Integrative Genomics Viewer (Robinson et al. 2011; Thorvaldsdóttir et al. 2013).

Mobile genetic elements in the genome of strain DSM 101085 were analyzed using IslandViewer 4 (http://www.pathogenomics.sfu.ca/islandviewer/; Dhillon et al. 2015; Bertelli et al. 2017). Identified regions that were predicted by at least two different implemented prediction methods were further examined for the presence of integrases and excisionases and accessory genes as main prerequisite. The presence of intact prophage and phage regions was further analyzed using PHASTER (Arndt et al. 2016). Detected (pro)phage regions were manually curated and when possible closely defined by predicting their attachment sites. For tree construction, analysis of both transposons and phages, VICTOR was used (Meier-Kolthoff and Göker 2017). All pairwise comparisons of the nucleotide sequences were conducted using the Genome-BLAST Distance Phylogeny method (Meier-Kolthoff et al. 2013) under settings recommended for prokaryotic viruses (Meier-Kolthoff and Göker 2017). The resulting intergenomic distances were used to infer a balanced minimum evolution tree with branch support via FASTME including SPR postprocessing (Lefort et al. 2015) for formula D0. Branch support was inferred from 100 pseudo-bootstrap replicates each. Trees were visualized with FigTree (http://tree.bio.ed.ac.uk/software/figtree/).

### Quantification of Toxins

TcdA and TcdB were quantified separately in at least three biological replicates in the culture supernatant after 48 h of growth using the TGC-E002-1 ELISA (tgcBIOMICS GmbH, Bingen, Germany). The expression of the subunits *cdtA* and *cdtB* of the binary toxin CDT was quantified by qPCR. For this, total RNA was extracted using the RNeasy Kit Mini (Qiagen, Hilden, Germany) according to the instructions of the manufacturer with the following modifications: cells were disrupted combining mechanical and enzymatical lysis by vigorously mixing with glass beads and lysozyme (15 mg/ml). Residual DNA was removed by two DNA digestion steps using the RNase-Free DNase Set (Qiagen) following the instructions of the manufacturer. Finally, the RNA was reverse transcribed into cDNA using the GoScript Reverse Transcription System (Promega, Madison, WI) according to the instructions of the manufacturer. Quantitative PCR was performed with 10 ng cDNA per sample in four biological and three technical replicates using the LightCycler 480 SYBR Green I Master Kit (Roche Diagnostics, Mannheim, Germany) according to the instructions of the manufacturer. Primers used for quantitative PCR were ACY CTT ACT TCC CCT GAA TAT GA (*cdtA*_F), AGA TAR GCT CCA GGA GAA CC (*cdtA*_R), TGC AGT TAA GTG GGA AGA TAG (*cdtB*_F), and GGA TAY GCT GCA ACT AAC GG (*cdtB*_R), respectively. Amplification efficiencies of the primer pairs were determined with serial DNA dilutions of strain DSM 27147 (R20291). Quantitative PCR was performed with the LightCycler 480 II system (Roche Diagnostics) as follows: initial denaturation at 95 °C for 5 min, 50 cycles at 95 °C for 10 s, 56 °C (*cdtA*) or 53 °C (*cdtB*) for 30 s, and 72 °C for 50 s.

### Metabolome and Exometabolome Analysis by GC-MS and HPLC-FLD

Sample preparation was performed as described previously (Zech et al. 2009; Reimer at al. 2014; Neumann-Schaal et al. 2015). Briefly, the precipitated cells were extracted by resuspension in methanol containing ribitol as internal standard, and lysis was enhanced by an ultrasonic bath followed by a methanol–water–chloroform extraction. The polar phase was transferred in a glass vial and dried under vacuum. The extracellular samples were prepared using 10 µl of the supernatant and 500 µl ethanol containing ribitol as internal standard. The samples were dried under vacuum as well. For GC-MS measurement, the dried samples were derivatized using a two-step protocol. In the first step, the samples were methoxymated with a methoxyamine hydrochloride solution (20 mg/ml) in pyridine. In the second step, a silylation was performed with *N*-methyl-*N*-(trimethylsilyl)-trifluoroacetamide.

Volatile compounds were measured after an ether extraction using 400 µl of the culture supernatant, 60 µl of a HPLC-grade sulfuric acid solution, and 600 µl of an internal standard solution of *o*-cresol with 200 µl *tert*-methylbutylether. The compounds were measured on a Agilent VF-WAXms column (0.25 mm × 30 m; Agilent, Santa Clara, CA) on a Thermo DSQ II gas chromatograph equipped with a liner and quadrupol mass spectrometer as described before (Neumann-Schaal et al. 2015).

Free amino acids were quantified on a 1260 Infinity HPLC system equipped with a fluorescence detector (Agilent Technologies, Waldbronn, Germany) and a Poroshell HPH-C18 separation column (4.6 × 100 mm, particle size 2.7 mm; Agilent Technologies). Samples were measured after precipitation of ammonium with a 1:1 dilution with sodium tetraphenylborate (250 mM). The HPLC method was used as described previously (Trautwein et al. 2016; Hofmann et al. 2018).

Analysis methods for each extracellularly detected compound are listed in supplementary table 3, Supplementary Material online. d-/l-Lactate were determined using the enzyme kit by UV detection (R-Biopharm, Darmstadt, Germany) following the instructions of the manufacturer.

## Results

### *Clostridioides difficile* DSM 101085 Harbors Several Transposons, Transposon-Like Elements, and Prophages

**Fig. 1.—:**
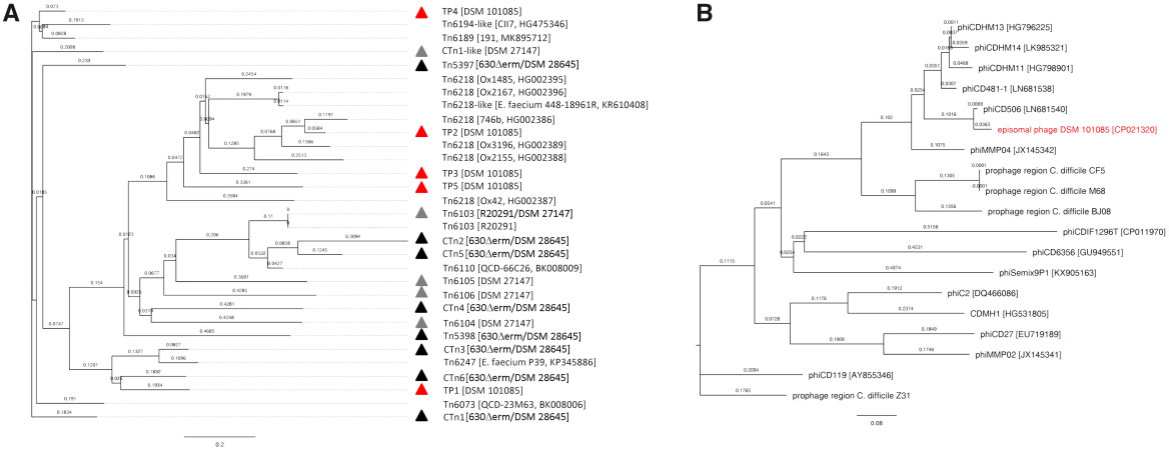
Transposons, transposon-like elements (*A*), and prophages of *C. difficile* DSM 101085 (*B*). For tree construction, analysis of both transposons and phages, VICTOR was used (Meier-Kolthoff and Göker 2017). All pairwise comparisons of the nucleotide sequences were conducted using the Genome-BLAST Distance Phylogeny method (Meier-Kolthoff et al. 2013) under settings recommended for prokaryotic viruses (Meier-Kolthoff and Göker 2017). The resulting intergenomic distances were used to infer a balanced minimum evolution tree with branch support via FASTME including SPR postprocessing (Lefort et al. 2015) for formula D0. Branch support was inferred from 100 pseudo-bootstrap replicates each. Trees were visualized with FigTree.

The presence of a Tn*6218*-like transposon (TP3) within the PaLoc of DSM 101085 prompted us to analyze the complete genome sequence of DSM 101085 for the presence of additional transposon-associated sequences and their phylogenetic relationship with other well-known *C. difficile* transposon sequences as found, for example, in the *C. difficile* strains 630 and R20291 (Brouwer et al. 2011). A total of five putative transposons and transposon-like regions were detected in the genome sequence of DSM 101085 (TP1–TP5, fig. 1*A*, table 1, and supplementary material 3.1, Supplementary Material online).

The two larger transposon-encoding sequences TP1 (CTn*6*-like) and TP4 (Tn*6194*-like) are more close to each other than to the others in term of size (20.8 and 21.6 kb, respectively) and gene organization/composition. They contain not only characteristic transposon gene clusters involved in regulation, integration, and excision but also accessory genes. The modules for accessory genes comprise putative genes for a replication initiation protein, antirestriction proteins (AdrA), a cell wall hydrolase, and varying numbers of transporter proteins. Compared with these sequences, the three remaining transposon-like elements TP2 (Tn*6218*-like), TP3, and TP5 (Tn*6218*-like) were smaller. Their genomic sizes ranged from 7.6 kb up to 12.4 kb and most of their gene products showed similarity to DNA-binding proteins with putative regulating functions or putative transporter proteins.

The search of similar transposons in other organisms using BlastN (transposons [taxid:2387]) allowed us to identify the presence of similar transposon-like elements not only in *Clostridioides* strains but also in some *Enterococci* and some other *Clostridiales*. TP1 (CTn*6*-like) showed similarity at the nucleotide level to a genomic region in *C. difficile* strain 630 (Sebaihia et al. 2006; Riedel et al. 2015; Dannheim, Riedel, et al. 2017), whereas TP4 (Tn*6194*-like) was found to be close to a Tn*6194*-like transposon from *C. difficile* strain CII7 (fig. 1*A*) and to not-annotated regions in *Roseburia intestinalis* and *Anaerostipes hadrus*. At the exception of the flanking genes in the chromosome, TP2 (Tn*6218*-like) was nearly identical to *C. difficile* transposon Tn*6218* in strain Ox746b, whereas TP3 (Tn*6218*-like) and TP5 were found to be partially similar in regard to gene content to Tn*6218*-like transposons in *C. difficile* and not-annotated regions of *Enterococci* (fig. 1*A*). Although transposons often participate in the spread of antibiotic resistance, none of the identified elements harbors a gene associated with antibiotic resistance.

Genome analysis revealed the presence of an episomal phiCD506-like bacteriophage which prompted us to analyze the genome of DSM 101085 for the presence of additional phage-associated genomic regions. Using PHASTER, we identified three putative prophage regions (table 1) in the chromosome of DSM 101085. Detailed analysis of the phage-tail (PT) region (table 1) showed only a partial phage genome: Several genes involved in tail structure and formation were identified including a tail sheath protein, a PT assembly protein, and a baseplate protein framed by putative regulatory genes (supplementary material 3.3, Supplementary Material online). In contrast, the prophage regions PR1 and PR2 consist of different gene clusters for putative proteins with conserved domains (supplementary material 3.3, Supplementary Material online) for DNA packaging, head and tail structure, as well as lysogeny or host lysis, and therefore can be regarded as functional prophages.

In both prophages PR1 and PR2 (supplementary material 3.3, Supplementary Material online), highly conserved structural genes for the composition of both phage heads and tails were identified as well as genes involved in replication. Additionally, both harbored genes for host cell lysis, specifically genes for putative holins with one and two transmembrane domains, respectively, and a putative amidase.

As a characteristic feature, both phage-encoding regions also contained genes involved in lysogeny, in particular for repressor-like proteins or proteins involved in recombination like integrases or recombinases. Interestingly, prophage PR2 also contained an *agr3* locus containing three genes (locus tags CDIF101085_03035-03037), namely *agrC* (pfam14501), *agrB* (pfam04647), and *agrD* (TIGR04223), notably found in *Clostridium* phage CDMH1. The genes present homologs in bacteria that are involved in the regulation of quorum sensing and virulence (Martin et al. 2013; Hargreaves et al. 2014).

The episomal bacteriophage-encoding element detected by genome sequencing was phylogenomically analyzed using VICTOR and found to be closely related to the myovirus phiCD506 (GenBank accession number LN681540, fig. 1*B*, supplementary material 3.2 , Supplementary Material online). It contains typical bacteriophage features including gene clusters for DNA packaging, host lysis, and characteristic head and tail structure. The only difference to phiCD506 is the 6-fold repetition of a small gene that might represent degenerated N-terminal parts of a helicase gene.

### Genetic Organization of the Toxin-Encoding Loci

**Fig. 2.—:**
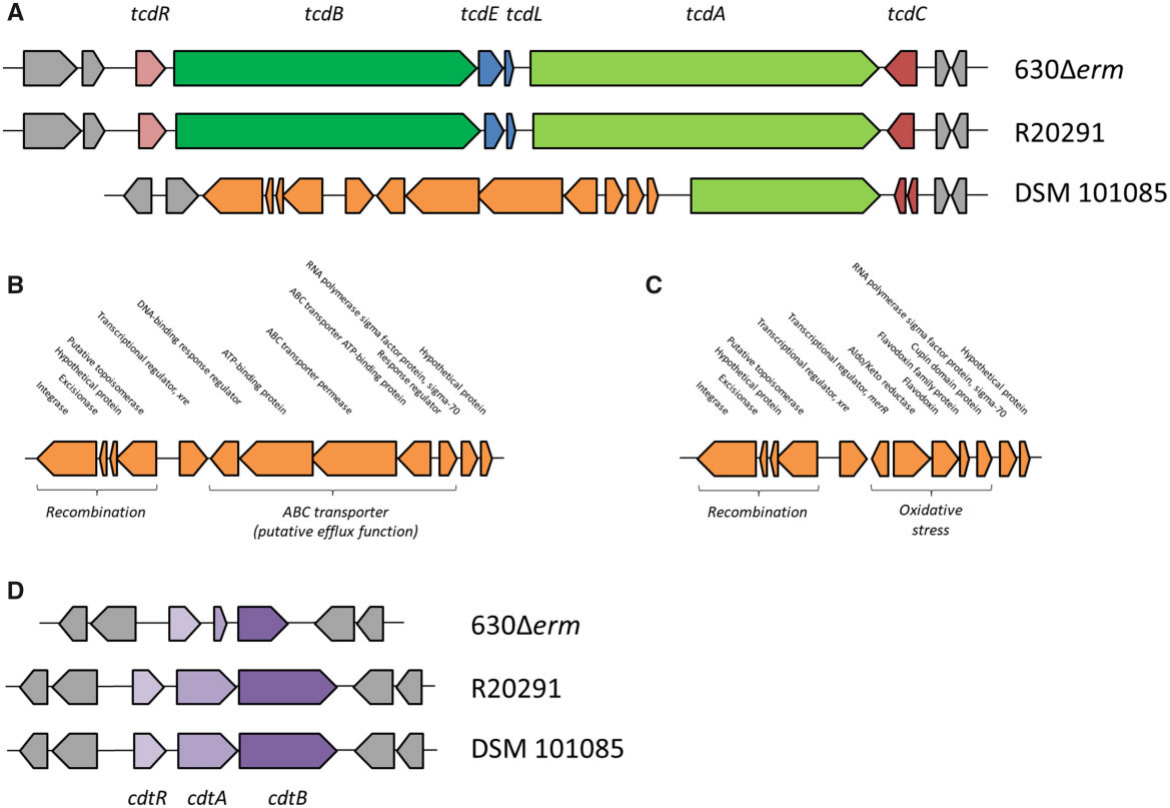
Genomic features of strain DSM 101085. (*A*) Comparison of PaLocs and flanking regions. The PaLocs of strain DSM 28645 (630Δ*erm*), DSM 27147 (R20291), and DSM 101085 were genomically compared. (*B*) Annotations of the PaLoc-associated transposon-like sequence in DSM 101085. (*C*) Annotations of the PaLoc-associated transposon-like sequence according Dingle et al. (2014). (*D*) Comparison of CdtLocs and its flanking regions. The CdtLocs of strain DSM 28645 (630Δ*erm*), DSM 27147 (R20291), and DSM 101085 were genomically compared.

The toxin pattern of DSM 101085 showed an atypical genetic organization of the PaLoc and canonical binary toxin-associated genes (CdtLoc), all encoded on the chromosome of the complete genome (fig. 2). Whereas the PaLoc typically contains the *tcdA* and *tcdB* gene encoding the toxin A and B, respectively (Braun et al. 1996), we only observed a truncated and disrupted pseudogene of *tcdA* (CDIF101085_00708) and no *tcdB*-encoding sequence in the genome of DSM 101085 (fig. 2*A*). Furthermore, a functional *tcdR* and *tcdC* gene involved in the regulation of the *tcdA* and *tcdB* gene, as well as *tcdE* and *tcdL* involved in the toxin secretion facilitation (Mani and Dupuy 2001; Matamouros et al. 2007; Govind and Dupuy 2012; Mehner-Breitfeld et al. 2018), could not be detected in the genome of strain DSM 101085 (fig. 2*A*). Only a disrupted *tcdC* gene (locus tags: CDIF101085_00710 and CDIF101085_00711) was detected (fig. 2*A*). This disrupted *tcdC* gene showed similarity to N-terminal truncated *tcdC* genes of hypervirulent NAP1 strains such as R20291 (Carter et al. 2011) and other clade 5 lineages, some considered not to be hypervirulent. Instead of *tcdR*, *tcdB*, *tcdE*, and a large genomic region, a Tn*6218*-like transposon-encoding sequence was detected upstream of the truncated *tcdA*. This sequence showed similarity to PaLoc-integrated transposon-sequences previously described for clade 3 isolates (Dingle et al. 2014; Chen et al. 2017; Elliott et al. 2017) and for potentially nontoxigenic strains of clade 5 (Elliott et al. 2009, 2014, 2017) (fig. 2). Compared with the transposon Tn*6218* that is inserted between *tcdE* and *tcdA* in clade 3 isolates (Dingle et al. 2014), the transposon of strain DSM 101085 showed a different gene content (fig. 2*B* and *C*). The Tn*6218*-like sequence of DSM 101085 contains a recombination module next to a module associated with putative ABC-transporter activity (fig. 2*B*) instead of a recombination and an oxidative stress related module identified in Tn*6218* transposons of clade 3 isolates (fig. 2*C*). In addition, a comparison of the 5′-end region of the *tcdA*-encoding sequence showed a high percentage of identity to sequence of strain *C. difficile* SE923 (GenBank accession number DQ914436, Geric Stare and Rupnik 2010).

Besides the atypical PaLoc, DSM 101085 also contains a complete CdtLoc encoding the genes *cdtR* (response regulator CdtR, CDIF101085_02724), *cdtA* (ADP-ribosyltransferase subunit, CDIF101085_02725), and *cdtB* (ADP-ribosyltransferase binding protein CdtB, CDIF101085_02726) (Perelle et al. 1997; Barth et al. 2004) as found, for example, in the hypervirulent strain R20291. In comparison, the CdtLoc of strain DSM 28645 (630Δ*erm*) only contains a nonfunctional CdtLoc with *cdtA*/*B* pseudogenes (fig. 2*D*).

The isolate *C. difficile* DSM 101085 was obtained from a patient who suffered from several recurrent infections and colonizations which likely contributed to the fatal outcome. The strain DSM 101085 was isolated ∼6 weeks after the previous strain (isolate 10) which was recently reported (Sachsenheimer et al. 2018). The complete genome of strain DSM 101085 (Riedel, Wetzel, et al. 2017) was compared with Illumina data published for the earlier isolate (isolate 10): Mapping the Illumina reads of this earlier isolate 10 (Sachsenheimer et al. 2018) onto the genome of strain DSM 101085 showed only a few differences between both genomes, especially very few single-nucleotide polymorphisms and no rearrangements. This striking resemblance between isolate 10 and DSM 101085 leaded us to conclude that DSM 101085 caused a relapse rather than representing a separate reinfection. The observed single-nucleotide polymorphisms (supplementary table 4, Supplementary Material online) might indicate short-term mutations and/or adaption within 6 weeks of colonization in the patient. Isolate 10 was only sequenced using Illumina technology resulting in a nonclosed genome assembly, which may not allow the detection of all sequence differences between both strains.

### Toxin Quantification Showed the Expression of the Binary Toxin

**Fig. 3.—:**
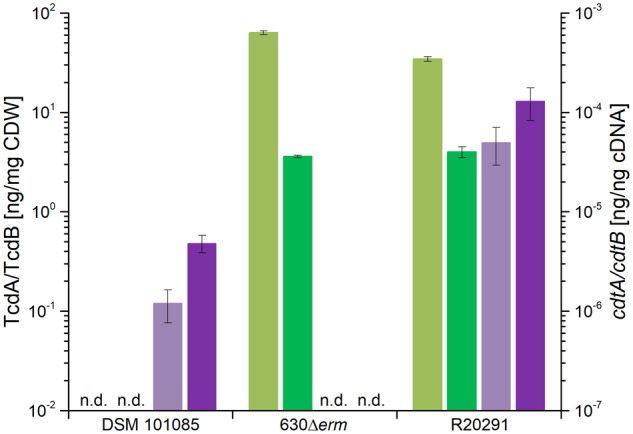
Quantification of toxins. Strains DSM 101085, DSM 27147 (R20291), and DSM 28645 (630Δ*erm*) were analyzed for the presence of the toxins TcdA (light green) and TcdB (dark green) by ELISA, as well as *cdtA* (light purple) and *cdtB* (dark purple) by qPCR.

*Clostridioides difficile* DSM 101085 was isolated from a patient with severe comorbidities who suffered from several severe CDI episodes before. This strain was isolated during an acute episode of CDI symptoms. Consequently, we analyzed toxin formation compared with chosen reference strains. For analysis of toxin formation of the three strains, we quantified TcdA and TcdB based on a commercially available ELISA system (fig. 3). We could confirm the absence of TcdA and TcdB in DSM 101085 and found that strain DSM 28645 (630Δ*erm*) produces higher TcdA amounts per cell dry weight compared with DSM 27147 (R20291), whereas TcdB levels were similar under the chosen conditions. We quantified the expression of the binary toxin-forming subunits *cdtA* and *cdtB* on the mRNA level. Whereas DSM 27147 (R20291) showed a higher gene expression than strain DSM 101085, binary toxin was not expressed by DSM 28645 (630Δ*erm*), in agreement with the absence of functional open reading frames in the CdtLoc (fig. 3). The sequences of CdtA and CdtB from strain DSM 101085 are 98% and 97% identical to the previously studied binary toxin of *C. difficile* strain CD196 (clade 2, RT027) and also to those of other hypervirulent *C. difficile* strains (supplementary material 4, Supplementary Material online, Schwan et al. 2009; He et al. 2010) with mainly conservative and semi-conservative exchanges leading to the assumption that the CDT itself would show similar effects in a cell culture system as previously described (Schwan et al. 2009).

### General Comparison of *C. difficile* DSM 101085 Metabolism to the Reference Strains

**Fig. 4.—:**
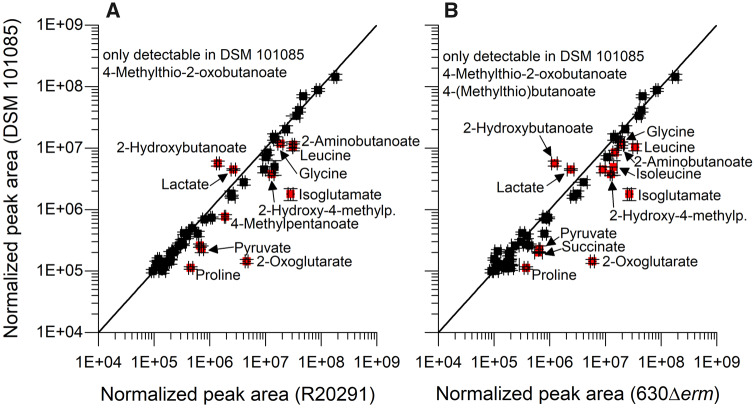
Comparison of logarithmized normalized peak areas between *C. difficile* DSM 101085 and the model strains. (*A*) Metabolites found in cell extracts after cultivation in casamino acids in comparison to the model strains DSM 28645 (630Δ*erm*) and (*B*) DSM 27147 (R20291) are shown. All strains were simultaneously grown until about 1/2 OD_max_. Values represent the average of four independent experiments. Error bars represent the standard deviation between the three experiments. Metabolites with an FC > 3 and a *P* value of >0.05 in a nonparametric Wilcoxon–Mann–Whitney test using the Benjamini–Hochberg correction are labeled. Detailed data are available as supplementary table 3, Supplementary Material online.

As for the exometabolome profile in the stationary phase (Riedel, Wetzel, et al. 2017), metabolome analysis of growing cells showed larger differences between DSM 101085 and DSM 28645 (630Δ*erm*) (Pearson correlation 0.9636) as well as between DSM 101085 and DSM 27147 (R20291) (Pearson correlation 0.9631), whereas the two reference strains were found to be highly similar (Pearson correlation 0.9972) at half-maximal growth (fig. 4). The exometabolome showed major differences especially concerning Stickland fermentation products (fig. 4). Distinct differences in the metabolome were observed in the reductive Stickland and central carbon metabolism associated fermentation. Proline was the only amino acid which was completely depleted from the medium at this stage of growth by all analyzed strains.

### Amino Acid Metabolism Is Altered in *C. difficile* DSM 101085

**Fig. 5.—:**
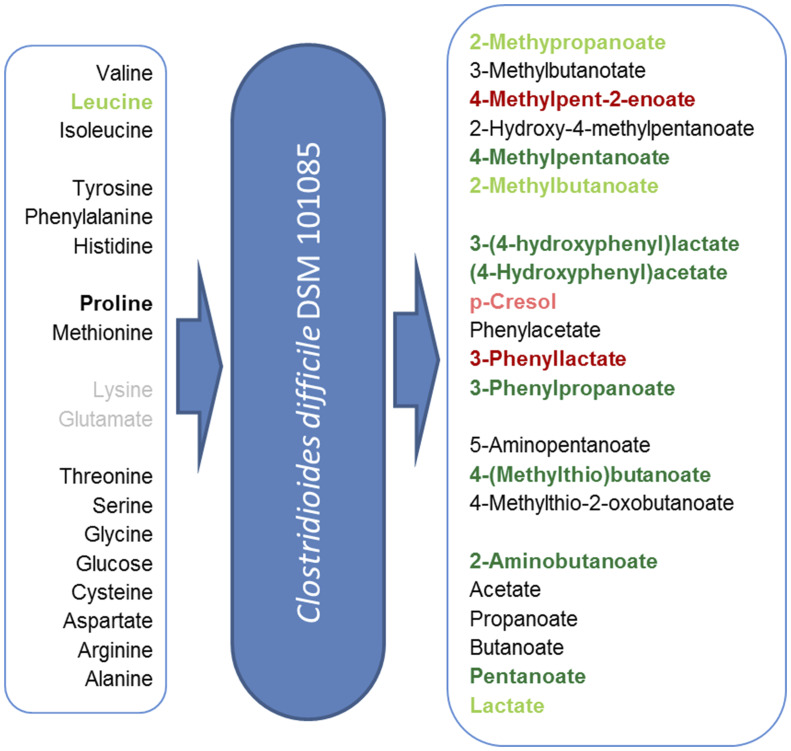
Specific formation of fermentation products and substrate usage. The relative abundances in the culture supernatant of four individual cultivations compared with the average of abundances in cultivations of the reference strains. Light green: >1.5-fold more abundant compared with one reference strain; dark green: >1.5-fold more abundant compared with both reference strains; light red: >1.5-fold less abundant compared with one reference strain; and dark red: >1.5-fold less abundant compared with both reference strains. Gray substrates were not used by one of the strains. Used methods for relative quantifications of each compound are listed in supplementary table 3, Supplementary Material online.

The methionine-based Stickland intermediate 4-methylthio-2-oxobutanoate was solely detectable intracellularly in DSM 101085 and the subsequent product 4-(methylthio)butanoate was solely detectable in DSM 101085 culture supernatants (figs. 4 and 5 and supplementary table 3, Supplementary Material online). Compared with other Stickland substrates, methionine usage seems to be subjected to larger differences between the isolates (Riedel, Wetzel, et al. 2017).

The oxidative Stickland product of tyrosine, (4-hydroxyphenyl)acetate, and its intermediate 3-(4-hydroxyphenyl)lactate were only detectable in DSM 101085 culture supernatants. Together with the decreased *p*-cresol content compared with DSM 27147 (R20291) (fold change [FC] 0.63), this points to a lower activity of the (4-hydroxyphenyl)acetate decarboxylase until half-maximal growth as tyrosine was consumed to the same extent. An alignment of the involved proteins (supplementary material 2.4, Supplementary Material online) showed only minor differences with mainly conservative exchanges. However, as structural information is not available, we cannot exclude an influence of enzyme activity.

Threonine metabolism was altered in DSM 101085 compared with the reference strains despite similar residual amounts in the medium. Although the abundance of the first degradation product, 2-oxobutanoate, was not altered itself, abundances of subsequent products such as 2-aminobutanoate (FC 0.36 [DSM 27147, R20291] and 0.59 [DSM 28645, 630Δ*erm*]), 2-hydroxybutanoate (FC 4.04 and 4.63), and pentanoate (FC 1.69 and 3.90) were changed which points toward a different preference of the available degradation pathways in different strains of *C. difficile* (Dannheim, Will, et al. 2017). Glycine levels were decreased in DSM 101085 (FC 0.63 and 0.61) which may be attributed to a reduced activity of the threonine aldolase pathway. The increased 2-hydroxybutanoate levels were most likely formed by the 2-hydroxyisocaproate dehydrogenase which is known to accept a wide variety of 2-oxo acids (Kim et al. 2006; Riedel, Wetzel, et al. 2017). A higher activity or better efficiency of this reductive Stickland pathway was also reflected by intracellular levels of the above-mentioned methionine-based pathway and by the leucine-derived product 4-methylpentanoate which was more abundant in the culture supernatant of DSM 101085 (FC 2.12 and 3.31). The leucine-derived intermediate product 4-methyl-pent-2-enoate was less abundant in the culture supernatant of DSM 101085 (FC 0.44 and 0.67). Taken together, strain DSM 101085 had more residual leucine in the culture supernatant compared with DSM 27147 but lower intracellular levels and has produced 2-fold more reductive Stickland product but secreted less intermediates (fig. 5). The reductive Stickland product of phenylalanine, 3-phenylpropanoate, was solely detectable in the culture supernatants of strain DSM 101085, whereas the intermediate 3-phenyllactate was less abundant (FC 0.29 and 0.36, fig. 5). An alignment of the proteins involved in the upper part of the reductive pathway showed no amino acid exchange between the two reference strains but several amino acid exchanges compared with DSM 101085 (supplementary material 2.4, Supplementary Material online). Although the exchanges are not directly part of the active site (Kim et al. 2006), they are partially located in proximity and may contribute to a higher efficiency of this pathway and to the conversion of the side substrate methionine. Exchanges in the lower part of the pathway are all conservative and even less frequent.

### Alteration of the Central Carbon Metabolism

Our analyses showed general differences in the central carbon metabolism and in important energy metabolism pathways (e.g., pyruvate, 2-oxoglutarate, succinate, and lactate) in the metabolism of DSM 101085 compared with the reference strains. Although pyruvate and 2-oxoglutarate were more abundant in both reference strains (pyruvate 0.32-fold [DSM 27147, R20291] and 0.35-fold [DSM 28645, 630Δ*erm*], 2-oxoglutarate 0.03- and 0.02-fold), succinate was only altered in strain DSM 28645 (630Δ*erm*, FC 0.32). Remarkably, we observed a lower intracellular isoglutamate concentration (0.06- and 0.07-fold) which serves as a compatible solute (Ruzicka and Frey 2007). The highest level of pentanoate was observed in DSM 101085 (fig. 5). This is in accordance with the increased 2-hydroxybutanoate levels serving as a precursor for pentanoate formation via propanoyl-CoA. Furthermore, we observed increased lactate levels (1.69- and 1.87-fold) in DSM 101085 even though lactate was only slightly increased in the culture supernatant (1.56- and 1.21-fold). As the used GC/MS method cannot differentiate between l- and d-lactate, we employed an enzymatic assay to confirm that only d-lactate was secreted in all three strains. The d-lactate dehydrogenase (corresponding to the locus tags CDIF630erm_01319-01321) with its three subunits shows a higher overall identity between the two reference strains (one amino acid exchange) compared with DSM 101085 (23 amino acid exchanges) (supplementary material 2.5, Supplementary Material online). This may contribute to both the activity of the enzymatic conversion and the efficiency of electron transfer via the two bifurcating subunits.

## Discussion

### DSM 101085 Evolved from a Hypervirulent Strain

The PaLoc of strain DSM 10185 only encodes an incomplete or fragmented PaLoc with a *tcdA* pseudogene and a disrupted *tcdC* gene. This PaLoc shows a completely different pattern than other so-called nontoxigenic *C. difficile* strains, where the PaLoc is replaced by a 115- or 75-bp fragment (Dingle et al. 2014). In regard to the missing well-known genes of a canonical PaLoc, a transposon with a high percentage of similarity to one of clade 3 isolates (Dingle et al. 2014; Chen et al. 2017; Peng et al. 2017) interrupted the classical PaLoc structure leading to a deletion of *tcdR*, *tcdB*, *tcdE*, *tcdL*, and most likely to the truncation of *tcdA* due to recombination events. The inserted transposon with a Tn*6218*-like character encodes for an ABC-type transporter with a putative efflux function which may contribute to a selective advantage compared with toxin expression. Similar PaLoc structures were described previously for different isolates (Rupnik 2008; Elliott et al. 2009, 2014; Geric Stare and Rupnik 2010). Moreover, we could experimentally prove that strain DSM 101085 did not produce the TcdA and TcdB with this specific PaLoc structure.

In contrast, we observed expression of the binary toxin-encoding subunit genes *cdtA* and *cdtB* in strain DSM 101085. Next to TcdA and TcdB, CDT is considered as a virulence-boosting factor in *C. difficile* (Aktories et al. 2018). Binary toxin-producing but TcdA and TcdB-negative strains are considered to be enterotoxic but did not cause infection in a hamster animal model (Geric et al. 2006). Other previous studies described cases of enteric symptoms and diarrhea caused by TcdA- and TcdB-negative strains (Geric et al. 2003; Eckert et al. 2015) and more recently CDT was shown to suppress protective colonic eosinophilia (Cowardin et al. 2016). Evidently, the role of CDT for *C. difficile* as well as for its host is still underestimated and needs further detailed investigation in future.

### *Clostridioides difficile* DSM 101085 Shows a More Efficient Utilization of Amino Acids

Changes in the central carbon metabolism and in important energy metabolism pathways may be explained by the regulation of the incomplete citric acid cycle in *C. difficile* where succinate and 2-oxoglutarate are not linked due to the absence of a functional oxoglutarate dehydrogenase (Dannheim, Will, et al. 2017). The decreased pyruvate levels were accompanied by increased lactate levels which points toward a higher activity of the lactate dehydrogenases even though extracellular lactate was only slightly increased. Pyruvate levels are of specific relevance in the context of the present study as Dubois et al. showed that pyruvate is one of the key metabolites linked to toxin production (Dubois et al. 2016).

For the branched chain amino acids and phenylalanine, we observed decreased secretion of the intermediates of the Stickland pathway. As the secretion of intermediates requires energy for transportation without allowing the reductive pathway to fulfill its metabolic role as reduction equivalent sink, this shows a more efficient usage of amino acids in the DSM 101085 strain. This is of particular interest as the patient was on a protein reduced diet and may be attributed to a long-term adaption of reduced availability of amino acids.

## Conclusions

*Clostridioides difficile* DSM 101085 was isolated from a patient who suffered from several recurrent infections and colonizations with *C. difficile* likely contributing to the fatal outcome caused by end-stage chronic kidney disease and congestive heart failure. A detailed view on the encoded toxins showed the presence of a complete CdtLoc and a nonfunctional PaLoc. Genomic analysis of DSM 101085 showed that the strain likely evolved from a previously hypervirulent strain which lost the virulence factors TcdA and TcdB but led to a recurrent infection in a patient with severe comorbidities and very susceptible for CDIs.

As several publications showed the connection between metabolism and toxin expression and as this patient was subjected to a very specific diet, we compared strain DSM 101085 with the *C. difficile* model strains DSM 28645 (630Δ*erm*) and DSM 27147 (R20291). The particular characteristics of strain *C. difficile* DSM 101085 showed a more efficient metabolization of amino acids and thus might represent a long-term adaptation to low-protein diet of the patient which may be still observable during growth in amino acid rich medium. This shows the high capability of *C. difficile* to adapt to specific nutritional environments and points toward a major role of diet in the context of recurrent CDIs.

## Supplementary Material

evaa072_Supplementary_Data
