# Supplementary material for: Characterization of Clostridioides difficile DSM 101085 with A−B−CDT+ Phenotype from a Late Recurrent Colonization
Source: Genome Biol Evol. 2020 Apr 17;12(5):566–77. doi: 10.1093/gbe/evaa072 (PMC7250501; doi:10.1093/gbe/evaa072)
Supplement: evaa072_Supplementary_Data [file evaa072_supplementary_data.zip › OP-GBEV200075_PECorr_CmtAttachmentsFolder_Supplementary_Material_2.docx]

# Supplementary Material 2 - General genome features of strain DSM 101085 and comparison with reference strains

The genome of DSM 101085 consists of a circular chromosome of 4,009,385 bp and an extrachromosomal bacteriophage of 36,250 bp (Supplementary Material 2.1 and 2.2). The chromosome contains 3,561 predicted coding sequences (CDS) with a coding density of 0.89 genes per kb and an average gene size of 912 bp. It includes 35 rRNAs and 90 tRNAs. The G+C content is 28.74%. The extrachromosomal element with episomal character encodes 57 CDS with a coding density of 1.57 genes per kb and an average gene size of 529 bp (Supplementary Material 2.1 and 2.2). This element has high similarity to the genome of bacteriophage phiCD506 (Sekulovic et al. 2014).

**Supplementary Material 2.1 General genome features of DSM 101085.**

| **Features** | **Chromosome** | **Extrachromosomal element** |
| --- | --- | --- |
| Size (bp) | 4,009,385 | 36,250 |
| G+C content (%) | 28.74 | 29.22 |
| Coding sequences | 3,561 | 57 |
| Coding density (genes/kb) | 0.89 | 1.57 |
| Average gene size (bp) | 912 | 529 |
| rRNAs | 35 | 0 |
| tRNAs | 90 | 0 |
| Accession No | CP021319.1 | CP021320.1 |

**
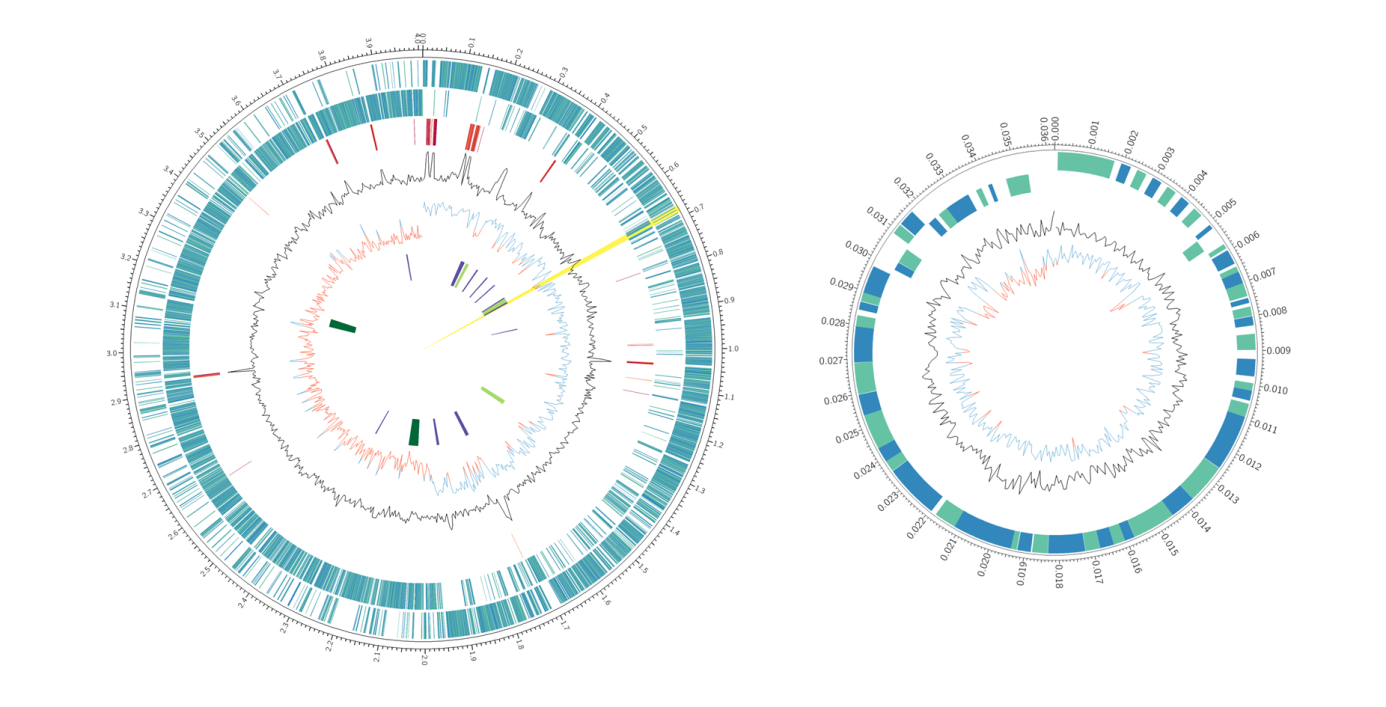
Supplementary Material 2.2. Genomic features of strain DSM 101085. Circular map of the chromosome (left) and the episomal bacteriophage (right).** From outer to inner circle: Forward strand, reverse strand, rRNA, GC content, GC-skew, genomic regions with putative mobile character (complete and intact prophages in dark green, incomplete prophages in light green, regions associated with transposable elements in purple). Circular plots were generated using Circos (Krzywinski et al. 2009).


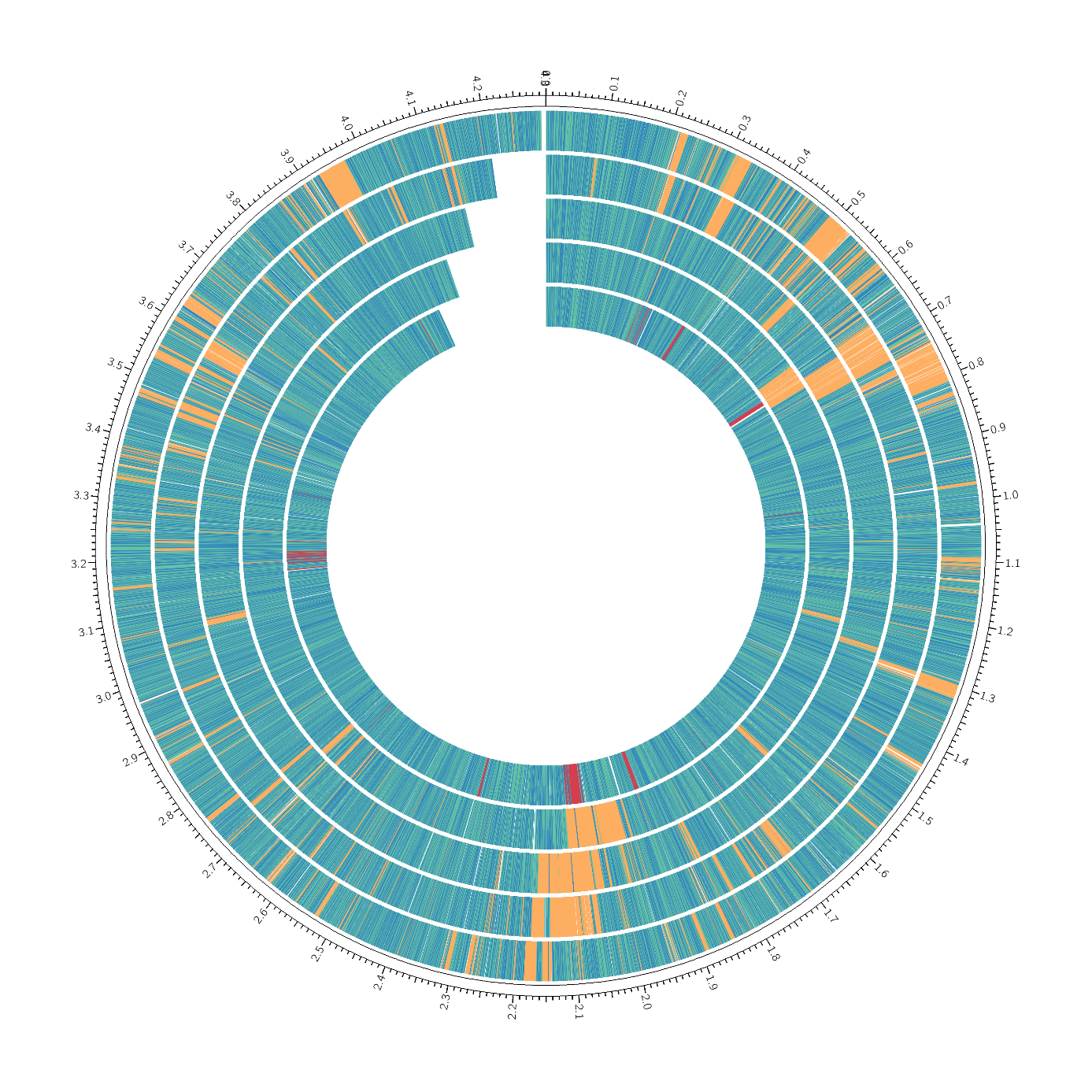


**Supplementary Material 2.3. Genome comparison of DSM 28645 (630Δ*erm*), DSM 27147 (R20291), DSM 29020, DSM 29747 and DSM 101085.** From outer to inner circle: DSM 28645 (630Δ*erm*, GenBank Acc. No. CP016318.1), DSM 27147 (R20291, GenBank Acc. No. CP029423.1), DSM 29020 (GenBank Acc. No. CP012325.1), DSM 29747 (GenBank Acc. No. CP019864.1) and DSM 101085 (GenBank Acc. No. CP021319.1). Genes present in all three genomes are shown in blue/green. Genes present only in DSM 101085 are marked in red, while genes present in the other four genomes, but not in DSM 101085 are shown in orange. Orthologues genes were determined with ProteinOrtho (Lechner et al. 2011).

**Supplementary Material 2.4. Alignment of the proteins involved in the upper part of the reductive pathway** of *C. difficile* 630∆*erm* (DSM 28645), *C. difficile* R20291 (DSM 27147) and *C. difficile* DSM 101085

CLUSTAL O(1.2.4) multiple sequence alignment

CDIF630erm_00522 MKILVFGARDYEEPVIKKWSEEHKDVQVDIYPENMTEENVVKAKGYDGISIQQTNYIDNP 60

CDIF27147_00502 MKILVFGARDYEEPVIKKWSEEHKDVQVDIYPENMTEENVVKAKGYDGISIQQTNYIDNP 60

CDIF101085_00489 MKILVFGARDYEEPVIKKWSEEHKDVQVDIYPENMTEENIVKAKGYDGISIQQTNYIDNP 60

***************************************:********************

CDIF630erm_00522 YIYETLKDAGVKVIASRTAGVDMIHFDLVNENGLIVTNVPSYSPNAIAELAVTQAMNLLR 120

CDIF27147_00502 YIYETLKDAGVKVIASRTAGVDMIHFDLVNENGLIVTNVPSYSPNAIAELAVTQAMNLLR 120

CDIF101085_00489 YIYETLKDAGVKVIASRTAGVDMIHFDLVNENGLIVTNVPSYSPNAIAELAVTQAMNLLR 120

************************************************************

CDIF630erm_00522 KTPLVKKKVCEGDYRWIAELLGTEVRSITVGVIGTGKIGATSAKLFKGLGANVIAFDQYP 180

CDIF27147_00502 KTPLVKKKVCEGDYRWIAELLGTEVRSITVGVIGTGKIGATSAKLFKGLGANVIAFDQYP 180

CDIF101085_00489 KTPLVKKKVCEGDYRWIAELLGTEVRSITVGVIGTGKIGATSAKLFKGLGANVIAFDQYP 180

************************************************************

CDIF630erm_00522 NSDLNDILTYKDSLEDLLKEADLITLHTPLLEGTKHMINKDTLAIMKDGAYIVNTGRGGL 240

CDIF27147_00502 NSDLNDILTYKDSLEDLLKEADLITLHTPLLEGTKHMINKDTLAIMKDGAYIVNTGRGGL 240

CDIF101085_00489 NSDLNDILTYKDSLEDLLKEADLITLHTPLLEGTKHMINKDTLAIMKDGAYIVNTGRGGL 240

************************************************************

CDIF630erm_00522 INTGDLIEALESGKIRAAALDTFETEGLFLNKKMNPGELTDPEINKLLSMEQVIFTHHLG 300

CDIF27147_00502 INTGDLIEALESGKIRAAALDTFETEGLFLNKKMNPGELTDPEINKLLSMEQVIFTHHLG 300

CDIF101085_00489 IKTEDLIEALESGKIRAAALDTFETEGLFLNKKMNPGELTDPEINKLLSMEQVIFTHHLG 300

*:* ********************************************************

CDIF630erm_00522 FFTSTAIENIVYSSLSSAVEVIKTGTATNRVN 332

CDIF27147_00502 FFTSTAIENIVYSSLSSAVEVIKTGTATNRVN 332

CDIF101085_00489 FFTSTAIENIVYSSLSSAVEVIKTGTATNRVN 332

********************************

CLUSTAL O(1.2.4) multiple sequence alignment

CDIF630erm_00523 MLLEGVKVVELSSFIAAPCCAKMLGDWGAEVIKIEPIEGDGIRVMGGTFKSPASDDENPM 60

CDIF27147_00503 MLLEGVKVVELSSFIAAPCCAKMLGDWGAEVIKIEPIEGDGIRVMGGTFKSPASDDENPM 60

CDIF101085_00490 MLLEGVKVVELSSFIAAPCCAKMLGDWGAEVIKIEPIEGDGIRVMGGTFKSPASDDENPM 60

************************************************************

CDIF630erm_00523 FELENGNKKGVSINVKSKEGVEILHKLLSEADIFVTNVRVQALEKMGIAYDQIKDKYPGL 120

CDIF27147_00503 FELENGNKKGVSINVKSKEGVEILHKLLSEADIFVTNVRVQALEKMGIAYDQIKDKYPGL 120

CDIF101085_00490 FELENGNKKGVSINVKSKEGVEILHKLLSEADIFVTNVRVQALEKMGIAYDQIKDKYPGL 120

************************************************************

CDIF630erm_00523 IFSQILGYGEKGPLKDKPGFDYTAYFARGGVSQSVMEKGTSPANTAAGFGDHYAGLALAA 180

CDIF27147_00503 IFSQILGYGEKGPLKDKPGFDYTAYFARGGVSQSVMEKGTSPANTAAGFGDHYAGLALAA 180

CDIF101085_00490 IFSQILGYGEKGPLKDKPGFDYTAYFARGGVSQSVMEKGTSPANTAAGFGDHYAGLALAA 180

************************************************************

CDIF630erm_00523 GSLAALHKKAQTGKGERVTVSLFHTAIYGMGTMITTAQYGNEMPLSRENPNSPLMTTYKC 240

CDIF27147_00503 GSLAALHKKAQTGKGERVTVSLFHTAIYGMGTMITTAQYGNEMPLSRENPNSPLMTTYKC 240

CDIF101085_00490 GSLAALHKKGQTGKGERVTVSLFHTAIYGMGTMITTAQYGNEMPLSRENPNSPLMTTYKC 240

*********.**************************************************

CDIF630erm_00523 KDGRWIQLALIQYNKWLGKFCKVINREYILEDDRYNNIDSMVNHVEDLVKIVGEAMLEKT 300

CDIF27147_00503 KDGRWIQLALIQYNKWLGKFCKVINREYILEDDRYNNIDSMVNHVEDLVKIVGEAMLEKT 300

CDIF101085_00490 KDGRWIQLALIQYNKWLGKFCKVINREYILEDDRYNNIDSMVNHVEDLVKIVGEAMLEKT 300

************************************************************

CDIF630erm_00523 LDEWSALLEEADLPFEKIQSCEDLLDDEQAWANDFLFKKTYDSGNTGVLVNTPVMFRNEG 360

CDIF27147_00503 LDEWSALLEEADLPFEKIQSCEDLLDDEQAWANDFLFKKTYDSGNTGVLVNTPVMFRNEG 360

CDIF101085_00490 LDEWSALLEEADLPFEKIQSCEDLLDDEQAWANDFLFKKTYDSGNTGVLVNTPVMFRNEG 360

************************************************************

CDIF630erm_00523 IKEYTPAPKVGQHTVEVLKSLGYDEEKINNFKDSKVVRY 399

CDIF27147_00503 IKEYTPAPKVGQHTVEVLKSLGYDEEKINNFKDSKVVRY 399

CDIF101085_00490 IKEYTPAPKVGQHTVEVLKSLGYDEEKINNFKDSKVVRY 399

***************************************

CLUSTAL O(1.2.4) multiple sequence alignment

CDIF630erm_00524 MYTMGLDIGSTASKGVILKNGEDIVASETISSGTGTTGPSRVLEKLYGKTGLAREDIKKV 60

CDIF101085_00491 MYTMGLDIGSTASKGVILKNGEDIVASETISSGTGTTGPSRVLEKLYGKTGLAREDIKKV 60

CDIF27147_00504 MYTMGLDIGSTASKGVILKNGEDIVASETISSGTGTTGPSRVLEKLYGKTGLAREDIKKV 60

************************************************************

CDIF630erm_00524 VVTGYGRMNYSDADKQISELSCHARGVNFIIPETRTIIDIGGQDAKVLKLDNNGRLLNFL 120

CDIF101085_00491 VVTGYGRMNYSDADKQISELSCHARGVNFIIPETRTIIDIGGQDAKVLKLDNNGRLLNFL 120

CDIF27147_00504 VVTGYGRMNYSDADKQISELSCHARGVNFIIPETRTIIDIGGQDAKVLKLDNNGRLLNFL 120

************************************************************

CDIF630erm_00524 MNDKCAAGTGRFLDVMAKIIEVDVSELGSISMNSQNEVSISSTCTVFAESEVISHLSENA 180

CDIF101085_00491 MNDKCAAGTGRFLDVMAKIIEVDVSELGSISMNSQNEVSISSTCTVFAESEVISHLSENA 180

CDIF27147_00504 MNDKCAAGTGRFLDVMAKIIEVDVSELGSISMNSQNEVSISSTCTVFAESEVISHLSENA 180

************************************************************

CDIF630erm_00524 KIEDIVAGIHTSVAKRVSSLVKRIGVQRNVVMVGGVARNSGIVRAMAREINTEIIVPDIP 240

CDIF101085_00491 KIEDIVAGIHTSVAKRVSSLVKRIGVQRNVVMVGGVARNSGIVRAMAREINTEIIVPDIP 240

CDIF27147_00504 KIEDIVAGIHTSVAKRVSSLVKRIGVQRNVVMVGGVARNSGIVRAMAREINTEIIVPDIP 240

************************************************************

CDIF630erm_00524 QLTGALGAALYAFDEAKESQKEVKNI 266

CDIF101085_00491 QLTGALGAALYAFDEAKESQKEVKNI 266

CDIF27147_00504 QLTGALGAALYAFDEAKESQKEVKNI 266

**************************

CLUSTAL O(1.2.4) multiple sequence alignment

CDIF630erm_00525 MSEKKEARVVINDLLAEQYANAFKAKEEGRPVGWSTSVFPQELAEVFDLNVLYPENQAAG 60

CDIF27147_00505 MSEKKEARVVINDLLAEQYANAFKAKEEGRPVGWSTSVFPQELAEVFDLNVLYPENQAAG 60

CDIF101085_00492 MSEKKEARVVINDLLAEQYANAFKAKEEGRPVGWSTSVFPQELAEVFDLNVLYPENQAAG 60

************************************************************

CDIF630erm_00525 VAAKKGSLELCEIAESKGYSIDLCAYARTNFGLLENGGCEALDMPAPDFLLCCNNICNQV 120

CDIF27147_00505 VAAKKGSLELCEIAESKGYSIDLCAYARTNFGLLENGGCEALDMPAPDFLLCCNNICNQV 120

CDIF101085_00492 VAAKKGSLELCEMAESKGYSIDLCAYARTNFGLLENGGCEALDMPAPDFLLCCNNICNQV 120

************:***********************************************

CDIF630erm_00525 IKWYENISRELDIPLIMIDTTFNNEDEVTQSRIDYIKAQFEEAIKQLEIISGKKFDPKKF 180

CDIF27147_00505 IKWYENISRELDIPLIMIDTTFNNEDEVTQSRIDYIKAQFEEAIKQLEIISGKKFDPKKF 180

CDIF101085_00492 IKWYENISRELDIPLIMIDTTFNNEDEVTQSRIDYIKAQFEEAIKQLEIISGKKFDPKKF 180

************************************************************

CDIF630erm_00525 EEVMKISAENGRLWKYSMSLPADSSPSPMNGFDLFTYMAVIVCARGKKETTEAFKLLIEE 240

CDIF27147_00505 EEVMKISAENGRLWKYSMSLPADSSPSPMNGFDLFTYMAVIVCARGKKETTEAFKLLIEE 240

CDIF101085_00492 EEVMKISAENGRLWKYSMSLPADSSPSPMNGFDLFTYMAVIVCARGKKETTEAFKLLIEE 240

************************************************************

CDIF630erm_00525 LEDNMKTGKSSFRGEEKYRIMMEGIPCWPYIGYKMKTLAKFGVNMTGSVYPHAWALQYEV 300

CDIF27147_00505 LEDNMKTGKSSFRGEEKYRIMMEGIPCWPYIGYKMKTLAKFGVNMTGSVYPHAWALQYEV 300

CDIF101085_00492 LEDNMKTGKSSFRGEEKYRIMMEGIPCWPYIGYKMKTLAKFGVNMTGSVYPHAWALQYEV 300

************************************************************

CDIF630erm_00525 NDLDGMAVAYSTMFNNVNLDRMTKYRVDSLVEGKCDGAFYHMNRSCKLMSLIQYEMQRRA 360

CDIF27147_00505 NDLDGMAVAYSTMFNNVNLDRMTKYRVDSLVEGKCDGAFYHMNRSCKLMSLIQYEMQRRA 360

CDIF101085_00492 NDLDGMAVAYSTMFNNVNLDRMTKYRVDSLIEGKCDGAFYHMNRSCKLMSLIQYEMQRRA 360

******************************:*****************************

CDIF630erm_00525 AEETGLPYAGFDGDQADPRAFTNAQFETRIQGLVEVMEERKKLNRGEI 408

CDIF27147_00505 AEETGLPYAGFDGDQADPRAFTNAQFETRIQGLVEVMEERKKLNRGEI 408

CDIF101085_00492 AEETGLPYAGFDGDQADPRAFTNAQFETRIQGLVEVMEERKKLNRGEI 408

************************************************

CLUSTAL O(1.2.4) multiple sequence alignment

CDIF27147_00506 MEAILSKMKEVVENPNAAVKKYKNETGKKAIGCFPVYCPEEIIHAAGMLPVGIWGGQTEL 60

CDIF630erm_00526 MEAILSKMKEVVENPNAAVKKYKSETGKKAIGCFPVYCPEEIIHAAGMLPVGIWGGQTEL 60

CDIF101085_00493 MEAILSKMKEVVENPNAAVKKYKSETGKKAIGCFPVYCPEEIIHAAGMLPVGIWGGQTEL 60

***********************.************************************

CDIF27147_00506 DLAKQYFPAFACSIMQSCLEYGLKGAYDELSGVIIPGMCDTLICLGQNWKSAVPHIKYIS 120

CDIF630erm_00526 DLAKQYFPAFACSIMQSCLEYGLKGAYDELSGVIIPGMCDTLICLGQNWKSAVPHIKYIS 120

CDIF101085_00493 DLAKQYFPAFACSIMQSCLEYGLKGAYDELSGVIIPGMCDTLICLGQNWKSAVPHIKYIS 120

************************************************************

CDIF27147_00506 LVHPQNRKLEAGVKYLISEYKGVKRELEEICGYEIEEAKIHESIEVYNEHRKTMRDFVEV 180

CDIF630erm_00526 LVHPQNRKLEAGVKYLISEYKGVKRELEEICGYEIEEAKIHESIEVYNEHRKTMRDFVEV 180

CDIF101085_00493 LVHPQNRKLEAGVKYLISEYKGVKRELEEICGYEIEEAKIHESIEVYNEHRKTMRDFVEV 180

************************************************************

CDIF27147_00506 AYKHSNTIKPSIRSLVIKSGFFMRKEEHTELVKDLIAKLNAMPEEVCSGKKVLLTGILAD 240

CDIF630erm_00526 AYKHSNTIKPSIRSLVIKSGFFMRKEEHTELVKDLIAKLNAMPEEVCSGKKVLLTGILAD 240

CDIF101085_00493 AYKHSNTIKPSIRSLVIKSGFFMRKEEHTKLVKDLIAKLNAMPEEVCSGKKVLLTGILAD 240

*****************************:******************************

CDIF27147_00506 SKDILDILEDNNISVVADDLAQETRQFRTDVPAGDDALERLARQWSNIEGCSLAYDPKKK 300

CDIF630erm_00526 SKDILDILEDNNISVVADDLAQETRQFRTDVPAGDDALERLARQWSNIEGCSLAYDPKKK 300

CDIF101085_00493 SKDILDILEDNNISVVADDLAQETRQFRTDVPAGDDALERLARQWSNIEGCSLAYDPKKK 300

************************************************************

CDIF27147_00506 RGSLIVDEVKKKDIDGVIFCMMKFCDPEEYDYPLVRKDIEDSGIPTLYVEIDQQTQNNEQ 360

CDIF630erm_00526 RGSLIVDEVKKKDIDGVIFCMMKFCDPEEYDYPLVRKDIEDSGIPTLYVEIDQQTQNNEQ 360

CDIF101085_00493 RGSLIVDEVKKKDIDGVIFCMMKFCDPEEYDYPLVRKDIEDSGIPTLYVEIDQQTQNNEQ 360

************************************************************

CDIF27147_00506 ARTRIQTFAEMMSLA 375

CDIF630erm_00526 ARTRIQTFAEMMSLA 375

CDIF101085_00493 ARTRIQTFAEMMSLA 375

***************

CLUSTAL O(1.2.4) multiple sequence alignment

CDIF630erm_00527 MLYNKEQELLRKAVRDFVSKELDTLPAEMDKTGVMPKELIKKLADAKFISSNIPEEYGGG 60

CDIF27147_00507 MLYNKEQELLRKAVRDFVSKELDTLPAEMDKTGVMPKELIKKLADAKFISSNIPEEYGGG 60

CDIF101085_00494 MLYNKEQELLRKAVRDFVSKELDTLPAEMDKTGVMPKELIKKLADAKFISSNIPEEYGGG 60

************************************************************

CDIF630erm_00527 GAGYVSYAIVMEEIARRCASTATFVTAGSSLASLPILYNGTEEQKQKYLKGIATGELIGA 120

CDIF27147_00507 GAGYVSYAIVMEEIARRCASTATFVTAGSSLASLPILYNGTEEQKQKYLKGIATGELIGA 120

CDIF101085_00494 GAGYVSYAIVMEEIARRCASTATFVTAGSSLASLPILYNGTEEQKQKYLKGIATGELIGA 120

************************************************************

CDIF630erm_00527 FGLTEPGAGSDAGGQQTTAELVGDHYILNGRKTFITNGPFCDVAIVIAVTDRSKGLRGTS 180

CDIF27147_00507 FGLTEPGAGSDAGGQQTTAELVGDHYILNGRKTFITNGPFCDVAIVIAVTDRSKGLRGTS 180

CDIF101085_00494 FGLTEPGAGSDAAGQQTTAELVGDHYILNGRKTFITNGPFCDVAIVIAVTDRSKGLRGTS 180

************.***********************************************

CDIF630erm_00527 AFIVESKWDGFSTGAHEDKMGIRGTETSDLIFENVKVPKENLLGKEGQGFKIAMGTLEVG 240

CDIF27147_00507 AFIVESKWDGFSTGAHEDKMGIRGTETSDLIFENVKVPKENLLGKEGQGFKIAMGTLEVG 240

CDIF101085_00494 AFIVESKWDGFSTGAHEDKMGIRGTETSDLIFENVKVPKENLLGKEGQGFKIAMGTLEVG 240

************************************************************

CDIF630erm_00527 RIGVAALALGIAQGALDEAVKYTKQRVQFGKPIAKFQNTQFTIADMETKVCAARGLVYDA 300

CDIF27147_00507 RIGVAALALGIAQGALDEAVKYTKQRVQFGKPIAKFQNTQFTIADMETKVCAARGLVYDA 300

CDIF101085_00494 RIGVAALALGIAQGALDEAVKYTKQRVQFGKPIAKFQNTQFTIADMETKVCAARGLVYDA 300

************************************************************

CDIF630erm_00527 AQKRDAGMRVAQESAMAKYYASEIANEVAYKALQLHGGYGFIKDYEIERMYRDARIVSIY 360

CDIF27147_00507 AQKRDAGMRVAQESAMAKYYASEIANEVAYKALQLHGGYGFIKDYEIERMYRDARIVSIY 360

CDIF101085_00494 AQKRDAGMRVAQESAMAKYYASEIANEVAYKALQLHGGYGFIKDYEIERMYRDARIVSIY 360

************************************************************

CDIF630erm_00527 EGTSEVQKMVISSNVLK 377

CDIF27147_00507 EGTSEVQKMVISSNVLK 377

CDIF101085_00494 EGTSEVQKMVISSNVLK 377

*****************

**Supplementary Material 2.5. Alignment of the three D-lactate dehydrogenase subunits** of *C. difficile* 630∆*erm* (DSM 28645), *C. difficile* R20291 (DSM 27147) and *C. difficile* DSM 101085.

CLUSTAL O(1.2.4) multiple sequence alignment

CDIF101085_01152 MEILTCIKQVPGTTSVEVDETTGVLKRDGVDSKMNPYDLYALETALRIKQEKKANLKVLS 60

CDIF630erm_01319 MEILTCIKQVPGTTSVEVDETTGVLKRDGVDSKMNPYDLYALETALRIKEDKKANLKVLS 60

CDIF27147_01177 MEILTCIKQVPGTTSVEVDETTGVLKRDGVDSKMNPYDLYALETALRIKEDKKANLKVLS 60

*************************************************::*********

CDIF101085_01152 MGPPQAKKVIEESFMMGADEGALISDRRFGGADVLATSYTISQGIKKMGKVDLIICGKQT 120

CDIF630erm_01319 MGPPQAKKVIEESFMMGADEGALISDRRFGGADVLATSYTISQGIKKMGKVDLIICGKQT 120

CDIF27147_01177 MGPPQAKKVIEESFMMGADEGALISDRRFGGADVLATSYTISQGIKKMGKVDLIICGKQT 120

************************************************************

CDIF101085_01152 TDGDTAQVGPEVAEFLDIPHVTNVTKLIEAKDESIVVEIDMPNDLQVCEIEYPCLITVEK 180

CDIF630erm_01319 TDGDTAQVGPEVAEFLDIPHVTNVTKLIEVKDESIVVEIDMPNDLQVCEIEYPCLITVEK 180

CDIF27147_01177 TDGDTAQVGPEVAEFLDIPHVTNVTKLIEVKDESIVVEIDMPNDLQVCEIEYPCLITVEK 180

*****************************.******************************

CDIF101085_01152 DIFQPRLPSFKLKLDTKDREIPVYSLDDFEDKDENNYGLNGSPTQVVRIFPPKPNTDKNI 240

CDIF630erm_01319 DIFQPRLPSFKLKLNTKDREIPVYSLDDFEDKNENNYGLNGSPTQVVRIFPPKPNTDKNI 240

CDIF27147_01177 DIFQPRLPSFKLKLNTKDREIPVYSLDDFEDKDENNYGLNGSPTQVVRIFPPKPNTDKNI 240

**************:*****************:***************************

CDIF101085_01152 VRGNADELSSALVNKLEELKLV 262

CDIF630erm_01319 VRGNADELSFALVNKLEELKLV 262

CDIF27147_01177 VRGNADELSFALVNKLEELKLV 262

********* ************

CLUSTAL O(1.2.4) multiple sequence alignment

CDIF630erm_01320 MSKIVVNQDKITDLKRILEICPFGAIEEKSGIVEISAGCKMCKLCVKSGPKGAFEFIESS 60

CDIF27147_01178 MSKIVVNQDKITDLKRILEICPFGAIEEKSGIVEISAGCKMCKLCVKSGPKGAFEFIESS 60

CDIF101085_01153 MSKIVVNQDKITDLKRVLEICPFGAIEEKSGVVEISAGCKMCKLCVKSGPKGAFEFVESS 60

****************:**************:************************:***

CDIF630erm_01320 KVQINKDEWRGIAVYVEHHNGNIHPVTYELIGKAREMASKIKQPVYCVFVGKDIKDKCSN 120

CDIF27147_01178 KVQINKDEWRGIAVYVEHHNGNIHPVTYELIGKAREMASKIKQPVYCVFVGKDIKDKCSN 120

CDIF101085_01153 KVQINKDEWRGIAVYVEHHNGNIHPVTYELIGKAREMASKIKQPVYCVFVGKDIKDKCNN 120

**********************************************************.*

CDIF630erm_01320 LISYGVDEVFVYDEDEFKDFRIEPYSKAIENFIDKIKPTIVLVGGTTLGRSLAPRLAARF 180

CDIF27147_01178 LISYGVDEVFVYDEDEFKDFRIEPYSKAIENFIDKIKPTIVLVGGTTLGRSLAPRLAARF 180

CDIF101085_01153 LISYGVDEVFVYDEDEFKDFRIEPYSKAIENFINKIKPTIVLVGGTTLGRSLAPRLAARF 180

*********************************:**************************

CDIF630erm_01320 RTGLTADCTILDIQSNTDLDQIRPAFGGNIMAHINTPNNRPQFATVRYKIFSAPEKIENT 240

CDIF27147_01178 RTGLTADCTILDIQSNTDLDQIRPAFGGNIMAHINTPNNRPQFATVRYKIFSAPEKIENT 240

CDIF101085_01153 RTGLTADCTILDIQSNTDLDQIRPAFGGNIMAHINTPNNRPQFATVRYKIFSVPEKIENA 240

****************************************************.******:

CDIF630erm_01320 TGKITLCKVEKKELKSKIKVLSVKEKNKEVGLEEAEVIVVASRAIKKQEDMEMIYKLADK 300

CDIF27147_01178 TGKITLCKVEKKELKSKIKVLSVKEKNKEVGLEEAEVIVVASRAIKKQEDMEMIYKLADK 300

CDIF101085_01153 TGKVTLCKIEKKDLSSKIKVLSVKEKNKEVGLEEAEVIVVASRAIKKQEDMEMMYKLANK 300

***:****:***:*.**************************************:****:*

CDIF630erm_01320 LNAQVAGTRPVIEAGWIDAKKQIGLSGRTVKPKLIITCGVSGAVQFVAGMQGADYIVAIN 360

CDIF27147_01178 LNAQVAGTRPVIEAGWIDAKKQIGLSGRTVKPKLIITCGVSGAVQFVAGMQGADYIVAIN 360

CDIF101085_01153 LNAQVAGTRPVIEAGWIDAKKQIGLSGRTVKPKLIITCGVSGAVQFVAGMQGADYIVAIN 360

************************************************************

CDIF630erm_01320 KDDKAPILDVAHLALIGDIYDIIPKLIEKIENNKDNNQKYMASAAK 406

CDIF27147_01178 KDDKAPILDVAHLALIGDIYDIIPKLIEKIENNKDNNQKYMASAAK 406

CDIF101085_01153 KDDKAPILDVAHLALIGDIYDIIPKLIEKIENNKDDNQKYMASAAK 406

***********************************:**********

CLUSTAL O(1.2.4) multiple sequence alignment

CDIF630erm_01321 MYKLIDKKDIDFLIDTCGEENVLVGSDINEDFSHDELGGIEKYPEVLVNVLETEQVSKIM 60

CDIF27147_01179 MYKLIDKKDIDFLIDTCGEENVLVGSDINEDFSHDELGGIEKYPEVLVNVLETEQVSKIM 60

CDIF101085_01154 MYKLVDKKDIDFLIDTCGEENVLVGSDINEDFSHDELGGIEKYPEVLVNVLETEQVSKIM 60

****:*******************************************************

CDIF630erm_01321 KYAYKNNIPVTPRGQGTGLVGAAVAINGGIMINLCKMNKILEVDYENLTLTVEPGVLLMT 120

CDIF27147_01179 KYAYKNNIPVTPRGQGTGLVGAAVAINGGIMINLCKMNKILEVDYENLTLTVEPGVLLMT 120

CDIF101085_01154 KYAYKNNIPVTPRGQGTGLVGAAVAINGGIMINLCKMNKILEVDYENLTLTVEPGVLLMT 120

************************************************************

CDIF630erm_01321 IGQYVQDRDLFYPPDPGEKSATIAGNINTNAGGMRAVKYGVTRDYVRGLEVVLPNGEIIN 180

CDIF27147_01179 IGQYVQDRDLFYPPDPGEKSATIAGNINTNAGGMRAVKYGVTRDYVRGLEVVLPNGEIIN 180

CDIF101085_01154 IGQYVQDRDLFYPPDPGEKSATIAGNINTNAGGMRAVKYGVTRDYVRGLEVVLPNGEVIN 180

*********************************************************:**

CDIF630erm_01321 VGGKVVKNSSGYSIKDLLVGSEGTLGIVTKAILKLLPLPKKSISLLIPFPDLSMAIETVP 240

CDIF27147_01179 VGGKVVKNSSGYSIKDLLVGSEGTLGIVTKAILKLLPLPKKSISLLIPFPDLSMAIETVP 240

CDIF101085_01154 VGGKVVKNSSGYSIKDLLVGSEGTLGIVTKAILKLLPLPKKSISLLVPFPDLSMAIETVP 240

**********************************************:*************

CDIF630erm_01321 KIIKSKSIPTAIEFMERDVILAAEEFLGKKFPDNTSDAYLLLTFDGNSTEDIEKEYEKVA 300

CDIF27147_01179 KIIKSKSIPTAIEFMERDVILAAEEFLGKKFPDNTSDAYLLLTFDGNSTEDIEKEYEKVA 300

CDIF101085_01154 KIIKSKSIPTATEFMERDVILAAEEFLGKKFPDNTSDAYLLLTFDGNSTEDIEKEYEKVA 300

*********** ************************************************

CDIF630erm_01321 NLCLENGALDVFISDTQERNDSIWSARGAFLEAIKASTTQMDECDVVVPRDKIAEFIRYT 360

CDIF27147_01179 NLCLENGALDVFISDTQERNDSIWSARGAFLEAIKASTTQMDECDVVVPRDKIAEFIRYT 360

CDIF101085_01154 NLCLENGALDVFISDTQERNDSIWSARGAFLEAIKAFTTQMDECDVVVPRDKIAEFIRYT 360

************************************ ***********************

CDIF630erm_01321 HELQDKLKIRIKSFGHAGDGNLHIYILKDGMDDNTWKIRLKETFDYMYKKSRELSGQVSG 420

CDIF27147_01179 HELQDKLKIRIKSFGHAGDGNLHIYILKDGMDDNTWKIRLKETFDYMYKKSRELSGQVSG 420

CDIF101085_01154 HELQDKLKIRIKSFGHAGDGNLHIYILKDGMDDNTWKIRLKETFDYMYKKSRELSGQVSG 420

************************************************************

CDIF630erm_01321 EHGIGYAKKEYLHESNSDAYMMLIKNIKLAFDPKNILNPGKIY 463

CDIF27147_01179 EHGIGYAKKEYLHESNSDAYMMLIKNIKLAFDPKNILNPGKIY 463

CDIF101085_01154 EHGIGYAKKEYLHESNSDAYMMLIKNIKLAFDPKNILNPGKIY 463

*******************************************

**References**

Lechner M, Findeiss S, Steiner L, Marz M, Stadler PF, et al. 2011. Proteinortho: detection of (co-)orthologs in large-scale analysis. BMC Bioinformatics. 12:124.

Krzywinski M, Schein J, Birol I, Connors J, Gascoyne R, et al. 2009. Circos: an information aesthetic for comparative genomics. Genome Res. 19:1639-1645.

Sekulovic O, Garneau JR, Néron A, Fortier LC. 2014. Characterization of temperate phages infecting *Clostridium difficile* isolates of human and animal origins. Appl Environ Microbiol. 80:2555-2563.
