# Supplementary material for: Characterization of Clostridioides difficile DSM 101085 with A−B−CDT+ Phenotype from a Late Recurrent Colonization
Source: Genome Biol Evol. 2020 Apr 17;12(5):566–77. doi: 10.1093/gbe/evaa072 (PMC7250501; doi:10.1093/gbe/evaa072)
Supplement: evaa072_Supplementary_Data [file evaa072_supplementary_data.zip › Supplementary_Material_1.docx]

**Supplementary Material 1 – Resequencing of R20291**

In the course of this study and analysis of the previously published genome of strain R20291 (He et al. 2010), we resequenced the widely used reference strain R20291 (DSM 27147) using a combination of SMRT and Illumina technology. Long-read assembly yielded one closed chromosome with a size of 4,204,902 bp (Suppl. Material 1.1). 3,676 coding sequences (CDS) were predicted with a coding density of 0.87 genes per kb and an average gene size of 941 bp, 35 rRNAs and 90 tRNAs. The G+C content is 28.87 % (Suppl. Material 1.1).

In comparison to the genome sequence previously published by He et al. (2010), the genome sequence of strain DSM 27147 (= NCTC 13366 = R20291) is 13,563 bp larger (Suppl. Material 1.1). Detailed analysis of the genomic regions associated with the larger size of the genome of DSM 27147 showed the complete resolution of rRNAs (35 versus 27), tRNA cluster (89 versus 0) and repeats. In addition, we determined two stretches of 100 non-coding ‘Ns’ each in the previously published sequence (He et al. 2010) which were completely resolved for DSM 27147. The ABC transporter genes frequently lost in R20291 isolates (Steglich et al. 2018) are present in the DSM 27147 strain. The genome sequence differences of DSM 27147 compared to the previously published R20291 (He et al. 2010) are shown in Suppl. Material 1.2.

**Supplementary Material 1.1. General genome features of R20291 (He et al. 2010) and the resequenced R20291-associated derivative DSM 27147.**

| **Features** | **R20291** | **DSM 27147** |
| --- | --- | --- |
| Size (bp) | 4,191,339 | 4,204,902 |
| G+C content (%) | 28.81 | 28.87 |
| Coding sequences | 3,521 | 3,676 |
| Coding density (genes/kb) | 0.84 | 0.87 |
| Average gene size (bp) | 973 | 941 |
| rRNAs | 27 | 35 |
| tRNAs | 0 | 89 |
| Accession No | FN545816.1 | CP029423.1 |

**Supplementary Material 1.2. INDEL and SNPs identified in DSM 27147 in comparison to R2091 (He et al. 2010).**

| **R20291**  **(FN545816.1)** | **indel/snp** | **DSM 27147 (CP029423.1)** | **Position information** |
| --- | --- | --- | --- |
| 15496 | ins 5391 bp | 15496 to 20886 | rRNA/tRNA cluster |
| 132816 to 132915 (Ns) | ins 1698 bp | 138207 to 139904 | rRNA/tRNA cluster |
| 132923 | C > CA | 139912 | NC/tRNA-Phe |
| 132939 | G > T | 139929 | NC/NC |
| 132955 | C > A | 139945 | NC/tRNA-Met |
| 132958 | T > G | 139948 | NC/tRNA-Met |
| 132959 | T > C | 139949 | NC/tRNA-Met |
| 143464 | A > AA | 150454 | NC/NC |
| 143507 to 143606 (Ns) | ins 6475 bp | 150498 to 156972 | rRNA/tRNA cluster |
| 143607 | C > A | 156972 | NC/16S rRNA |
| 143630 | C > T | 156995 | NC/16S rRNA |
| 143637 | C > A | 157002 | NC/16S rRNA |
| 143668 | T > A | 157033 | NC/16S rRNA |
| 143672 | C > T | 157037 | NC/16S rRNA |
| 206402 | A > AA | 219767 | NC/NC |
| 308259 to 308453 | reverse complement | 321625 to 321819 | flgB flagellar switch region |
| 581483 | A > AA | 594849 | NC/NC |
| 581490 | A > AA | 594857 | NC/NC |
| 581497 | A > AA | 594865 | NC/NC |
| 593914 | ins 70bp | 607283 to 607352 | NC/NC (repetitive) |
| 672194 | AA > A | 685631 | NC/NC |
| 752573 to 752591 | del 19bp | 766006 | NC/NC (repetitive) |
| 825955 | ins 149bp | 839373 to 839521 | CDR20291_0667 (pseudogene)/NC (repetitive) |
| 1564436 | AA > A | 1578002 | NC/NC |
| 1568676 | C > A | 1582241 | CDR20291_1323/R20291_01496 |
| 1578173 | TT > T | 1591738 | NC/NC |
| 1578208 | A > AA | 1591772 | NC/NC |
| 1592813 | A > T | 1606378 | NC/NC |
| 1864420 | T > TT | 1877986 | CDR20291_1576/NC |
| 1899603 | AA > A | 1913169 | NC/NC |
| 2235742 | TT > T | 2249307 | CDR20291_1913/R20291_02147 |
| 2262064 | A > AA | 2275630 | NC/NC |
| 2264190 | TT > T | 2277755 | NC/NC |
| 2298115 | T > TT | 2311679 | NC/NC |
| 2361948 | C > A | 2375513 | NC/NC |
| 2361960 | A > AA | 2375525 | NC/NC |
| 2367947 | T > TT | 2381513 | NC/NC |
| 2578163 | TT > T | 2591730 | NC/NC |
| 2674748 | TT > T | 2688314 | NC/NC |
| 2680791 | T > TT | 2694356 | NC/NC |
| 2772183 | TT > T | 2785749 | CDR20291_2368_frameshift/R20291_02637 |
| 3067060 | T > TT | 3080625 | 16S rRNA |
| 3067095 | T > TT | 3080661 | 16S rRNA |
| 3077993 | AA > A | 3091560 | NC/NC |
| 3162104 | TT > T | 3175670 | NC/NC |
| 3361922 | AA > A | 3375487 | NC/NC |
| 3846386 | AA > A | 3859950 | NC/NC |


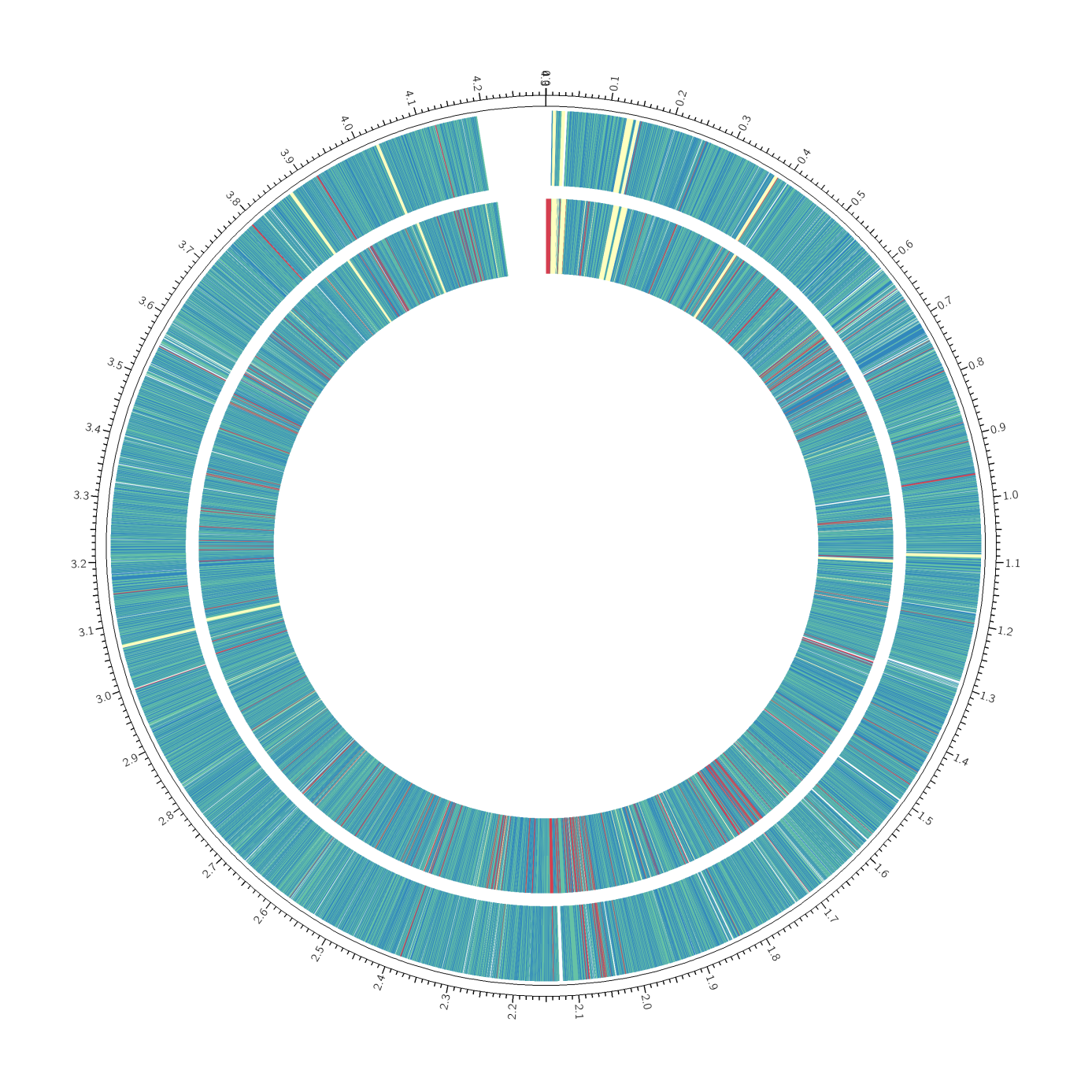


**Supplementary Material 1.3. Genome comparison of R20291 (He et al. 2010) and DSM 27147.** From outer to inner circle: R20291 (GenBank Acc. No. FN545816.1), strain DSM 27147 (R20291, GenBank Acc. No. CP029423.1). Orthologues genes were determined with ProteinOrtho (Lechner et al. 2011) and are shown in blue/green in comparison to strain R20291. Whereas RNAs (rRNA, tRNA and ncRNA) are shown in yellow, non-orthologues genes are shown in red.

**References**

He M, Sebaihia M, Lawley TD, Stabler RA, Dawson LF, et al. 2010. Evolutionary dynamics of *Clostridium difficile* over short and long time scales. Proc. Natl. Acad. Sci. U S A. 107:7527-7532.

Lechner M, Findeiss S, Steiner L, Marz M, Stadler PF, et al. 2011. Proteinortho: detection of (co-)orthologs in large-scale analysis. BMC Bioinformatics. 12:124.

Steglich M, Hofmann JD, Helmecke J, Sikorski J, Spröer C, Riedel T, et al. 2018. Convergent loss of ABC transporter genes from *Clostridioides difficile* genomes is associated with impaired tyrosine uptake and *p*-cresol production. Front Microbiol. 9:901.
