# Supplementary material for: Characterization of Clostridioides difficile DSM 101085 with A−B−CDT+ Phenotype from a Late Recurrent Colonization
Source: Genome Biol Evol. 2020 Apr 17;12(5):566–77. doi: 10.1093/gbe/evaa072 (PMC7250501; doi:10.1093/gbe/evaa072)
Supplement: evaa072_Supplementary_Data [file evaa072_supplementary_data.zip › Supplementary_Material_3.docx]

**Supplementary Material 3 – Transposons, transposon-like elements and prophages**

**
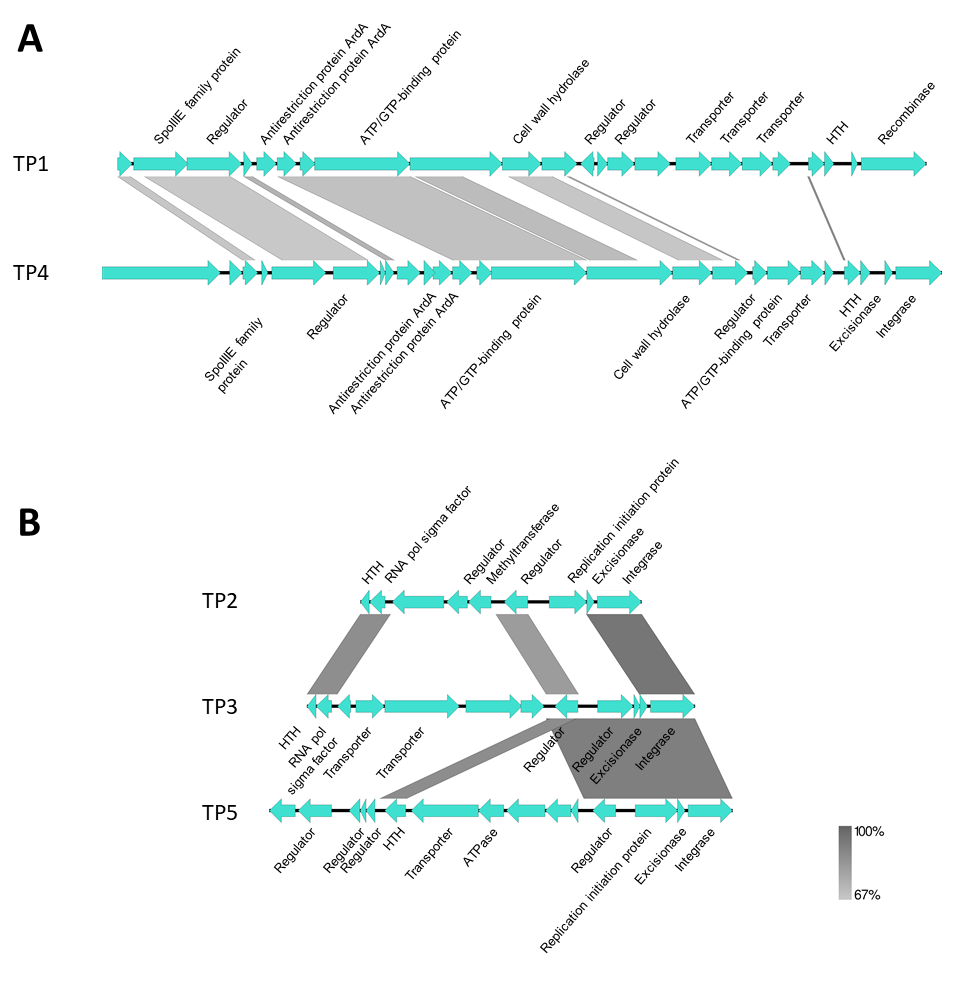
**

**Supplementary Material 3.1. Comparison of detected large (A) and small (B) transposons and transposon-like elements.** Synteny plot at the nucleotide level was constructed and visualized with Easyfig (Sullivan et al. 2011).

**
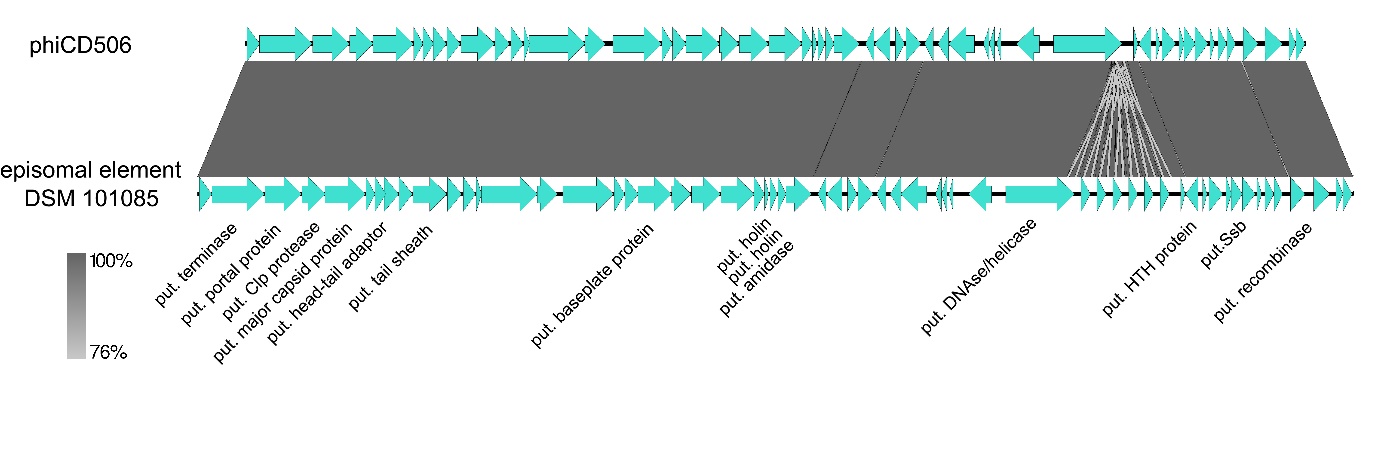
**

**Supplementary Material 3.2. Comparison of the episomal element of DSM 101085 with *C. difficile* phage phiCD506 on nucleotide level.** Synteny plot was constructed and visualized with Easyfig (Sullivan et al. 2011).

**Supplementary Material 3.3. Putative functions of prophage-encoded genes.**

| **Phage ID** | **Locus tag** | **Putative function** | **Conserved domain** |
| --- | --- | --- | --- |
| **PT** | CDIF101085_01336 | tail sheath protein | Phage sheath 1 pfam04984 |
|  | CDIF101085_01338 | a phage  tail assembly protein | Phage_TAC_5 pfam08890 |
|  | CDIF101085_01345 | baseplate protein | Baseplate_J pfam04865 |
| **PR1** | CDIF101085_02001 | DNA binding protein | HTH domain |
|  | CDIF101085_02010 | repressor | COG3682 |
|  | CDIF101085_02012 | DNA binding protein | HTH domain |
|  | CDIF101085_02016 | amidase | Amidase_3 pfam01520 |
|  | CDIF101085_02017 | holin | 2 TMDs |
|  | CDIF101085_02018 | holin | 1 TMD |
|  | CDIF101085_02023 | baseplate protein | Baseplate_J pfam04865 |
|  | CDIF101085_02031 | tail tape measure protein | tape_meas_TP901 TIGR01760 |
|  | CDIF101085_02033 | tail assembly protein | Phage_TAC_5 pfam08890 |
|  | CDIF101085_02035 | tail sheath protein | Phage sheath 1 pfam04984 |
|  | CDIF101085_02046 | portal protein | Phage_prot_gp6 pfam05133 |
|  | CDIF101085_02047 | terminase large subunit | Terminase_6 pfam03237 |
|  | CDIF101085_02048 | terminase small subunit | Phage_terminase pfam10668 |
|  | CDIF101085_02055 | endonuclease | RusA COG4570 |
|  | CDIF101085_02076 | Erf protein | ERF pfam04404 |
|  | CDIF101085_02082 | DNA binding protein | HTH domain |
|  | CDIF101085_02085 | anti-repressor | COG3561 |
|  | CDIF101085_02090 | DNA binding protein | HTH domain |
|  | CDIF101085_02092 | DNA binding protein | HTH domain |
|  | CDIF101085_02093 | integrase | INT_ICEBs1_C_like cd01189 |
| **PR2** | CDIF101085_03039 | amidase | Amidase_3 pfam01520 |
|  | CDIF101085_03040 | holin | 2 TMDs |
|  | CDIF101085_03041 | holin | 1 TMD |
|  | CDIF101085_03048 | baseplate protein | Baseplate_J pfam04865 |
|  | CDIF101085_03053 | tail tape measure protein | tape_meas_nterm TIGR02675 |
|  | CDIF101085_03061 | tail assembly protein | Phage_TAC_5 pfam08890 |
|  | CDIF101085_03063 | tail sheath protein | Phage sheath 1 pfam04984 |
|  | CDIF101085_03077 | portal protein | portal_SPP1 TIGR01538 |
|  | CDIF101085_03078 | terminase subunit | Terminase_3 pfam04466 |
|  | CDIF101085_03079 | terminase small subunit | YjcR COG5484 |
|  | CDIF101085_03087 | anti-repressor protein | COG3617 |
|  | CDIF101085_03091 | endonuclease | RusA pfam05866 |
|  | CDIF101085_03100 | single strand binding protein | ssb TIGR00621 |
|  | CDIF101085_03101 | DNA replication protein | DnaD COG3935 |
|  | CDIF101085_03102 | Erf protein | ERF pfam04404 |
|  | CDIF101085_03108 | anti-repressor protein | COG3617 |
|  | CDIF101085_03112 | DNA binding protein | HTH domain |
|  | CDIF101085_03113 | recombinase | PinE COG1961 |

**References**

Sullivan MH, Petty NK, Beatson SA. 2011. Easyfig: a genome comparison visualizer. Bioinformatics. 27:1009-1010.
