# Supplementary material for: Characterization of Clostridioides difficile DSM 101085 with A−B−CDT+ Phenotype from a Late Recurrent Colonization
Source: Genome Biol Evol. 2020 Apr 17;12(5):566–77. doi: 10.1093/gbe/evaa072 (PMC7250501; doi:10.1093/gbe/evaa072)
Supplement: evaa072_Supplementary_Data [file evaa072_supplementary_data.zip › Supplementary_Material_4.docx]

**Supplementary Material 4. Alignment of the CdtA and CdtB** of *C. difficile* DSM 101085 and CD196.

**CdtA**

CLUSTAL O(1.2.4) multiple sequence alignment

CD196 MKKFRKHKRISNCISILLILYLTLGGLLPNNIYAQDLQSYSEKVCNTTYKAPIERPEDFL 60

DSM101085 MKKFRKHKSISNCISILLILYLTLGSLLPNNIYAQDLQSYSEKVCNTTYKAPIERPEDFL 60

******** ****************.**********************************

CD196 KDKEKAKEWERKEAERIEQKLERSEKEALESYKKDSVEISKYSQTRNYFYDYQIEANSRE 120

DSM101085 KDKERAKEWERKEAERIEQKLERSEKEALESYKKDSVEINKYSQTRNYFYDYQIEANSRE 120

****:**********************************.********************

CD196 KEYKELRNAISKNKIDKPMYVYYFESPEKFAFNKVIRTENQNEISLEKFNEFKETIQNKL 180

DSM101085 KEYRELRNAISKNKIDKPMYVYYFESPEKFAFNKVIRTENQNEISLEKFNEFKETIQNKL 180

***:********************************************************

CD196 FKQDGFKDISLYEPGKGDEKPTPLLMHLKLPRNTGMLPYTNTNNVSTLIEQGYSIKIDKI 240

DSM101085 FKQDGFKEISLYEPGKGDEEPTPLLMHLKLPRNTGMLPYTNTNNVSTLIEQGYSIKIDKI 240

*******:***********:****************************************

CD196 VRIVIDGKHYIKAEASVVSSLDFKDDVSKGDSWGKANYNDWSNKLTPNELADVNDYMRGG 300

DSM101085 VRIVIDGKHYIKAEASVVSSLDFKDDVSKGDSWGKANYNDWSNKLTPNELADVNDYMRGG 300

************************************************************

CD196 YTAINNYLISNGPVNNPNPELDSKITNIENALKREPIPTNLTVYRRSGPQEFGLTLTSPE 360

DSM101085 YTAINNYLISNGPVNNPNPELDSKITNIENALKREPIPTNLTVYRRSGPQEFGLTLTSPE 360

************************************************************

CD196 YDFNKLENIDAFKSKWEGQALSYPNFISTSIGSVNMSAFAKRKIVLRITIPKGSPGAYLS 420

DSM101085 YDFNKPENIDAFKSKWEGQTLSYPNFISTSIGSVNMSAFAKRKIVLRITIPKGSPGAYLS 420

***** *************:****************************************

CD196 AIPGYAGEYEVLLNHGSKFKINKIDSYKDGTITKLIVDATLIP 463

DSM101085 AIPGYAGEYEVLLNHGSKFKISKIDSYKDGAITKLIVDATLIP 463

*********************.********:************

**CdtB**

CD196 MKIQMRNKKVLSFLTLTAIVSQALVYPVYAQTSTSNHSNKKKEIVNEDILPNNGLMGYYF 60

DSM101085 MKVQMRNKKVLSFLTLTAIVSQALAYPVYAQTSTSSHSDNKKEIINEDILTNNGLMGYYF 60

**:*********************.**********.**::****:***** *********

CD196 TDEHFKDLKLMAPIKDGNLKFEEKKVDKLLDKDKSDVKSIRWTGRIIPSKDGEYTLSTDR 120

DSM101085 TDEHFKDLKLMAPIKDGNLKFEEKKVDKLLNKDKSNVKSIRWTGRIIPSKDGEYTLSTDR 120

******************************:****:************************

CD196 DDVLMQVNTESTISNTLKVNMKKGKEYKVRIELQDKNLGSIDNLSSPNLYWELDGMKKII 180

DSM101085 DDILMQVNNESTISNTLKVNMKKGKEYKFRIELQDKNLGSIDNLSSPNLYWELDGIKKII 180

**:*****.*******************.**************************:****

CD196 PEENLFLRDYSNIEKDDPFIPNNNFFDPKLMSDWEDEDLDTDNDNIPDSYERNGYTIKDL 240

DSM101085 PAENLFLRDYSNIEKNDPFIPNNNFFDPRLMSDWEDEDLDTDNDNIPDSYERNGYTIKDL 240

* *************:************:*******************************

CD196 IAVKWEDSFAEQGYKKYVSNYLESNTAGDPYTDYEKASGSFDKAIKTEARDPLVAAYPIV 300

DSM101085 IAVKWEDSFAEQGYKKYVSNYLESNTAGDPYTDYEKASGSFDKAIKTEARDPLVAAYPIV 300

************************************************************

CD196 GVGMEKLIISTNEHASTDQGKTVSRATTNSKTESNTAGVSVNVGYQNGFTANVTTNYSHT 360

DSM101085 GVGMEKLIISTNEHASTDQGKTVSRATTNSKTESNTAGVSVNVGYQNGFTANVTTNYSHT 360

************************************************************

CD196 TDNSTAVQDSNGESWNTGLSINKGESAYINANVRYYNTGTAPMYKVTPTTNLVLDGDTLS 420

DSM101085 TDNSTAVQDSNGESWNTGLSINKGESAYINANVRYYNTGTAPMYKVTPTTNLVLDGDTLS 420

************************************************************

CD196 TIKAQENQIGNNLSPGDTYPKKGLSPLALNTMDQFSSRLIPINYDQLKKLDAGKQIKLET 480

DSM101085 TIKAQENQIGNNLSPGDTYPKKGLSPLALNTMDQFSSRLIPINYDQLKKLDAGKQIKLET 480

************************************************************

CD196 TQVSGNFGTKNSSGQIVTEGNSWSDYISQIDSISASIILDTENESYERRVTAKNLQDPED 540

DSM101085 TQVSGNFGTKNSSGQIVTEGNSWSDYISQIDSISASIILDTENESYERRVTAKNLQNPED 540

********************************************************:***

CD196 KTPELTIGEAIEKAFGATKKDGLLYFNDIPIDESCVELIFDDNTANKIKDSLKTLSDKKI 600

DSM101085 KTPELTIGEAIEKAFGATKKDGLLYFNDIPIDESCVELIFDDNTANKIKDSLKTLSDKKI 600

************************************************************

CD196 YNVKLERGMNILIKTPTYFTNFDDYNNYPSTWSNVNTTNQDGLQGSANKLNGETKIKIPM 660

DSM101085 YNVKLERGMNILIKTPTYFTNFDDYNNYPSTWSNVNTTNQDGLQGSANKLNGETKIKIPM 660

************************************************************

CD196 SELKPYKRYVFSGYSKDPLTSNSIIVKIKAKEEKTDYLVPEQGYTKFSYEFETTEKDSSN 720

DSM101085 SKLKPYKRYVFSGYSKDPLTSNSIIVKIKAKEEKTDYLVPEQGYTKFSYEFETTEKDSSN 720

*:**********************************************************

CD196 IEITLIGSGTTYLDNLSITELNSTPEILDEPEVKIPTDQEIMDAHKIYFADLNFNPSTGN 780

DSM101085 IEITLIGSGTTYLDNLSITELNSTPEILNEPEVKIPTDQEIIDAHKIYSADLNFNPSTGN 780

****************************:************:****** ***********

CD196 TYINGMYFAPTQTNKEALDYIQKYRVEATLQYSGFKDIGTKDKEMRNYLGDPNQPKTNYV 840

DSM101085 AYINGMYFTPTQTNKEALDYIQKYRVEATLQYSGFKDIGTKDKEMRNYLGDPNQPKTNYV 840

:*******:***************************************************

CD196 NLRSYFTGGENIMTYKKLRIYAITPDDRELLVLSVD 876

DSM101085 NLRSYFTGGENIMTYKKLRIYAITPDDRELLVLSVD 876

************************************
